# Supplementary figures and images for: A non-transcriptional function of Yap regulates the DNA replication program in Xenopus laevis
Source: eLife. 2022 Jul 15;11:e75741. doi: 10.7554/eLife.75741 (PMC9328763; doi:10.7554/eLife.75741)

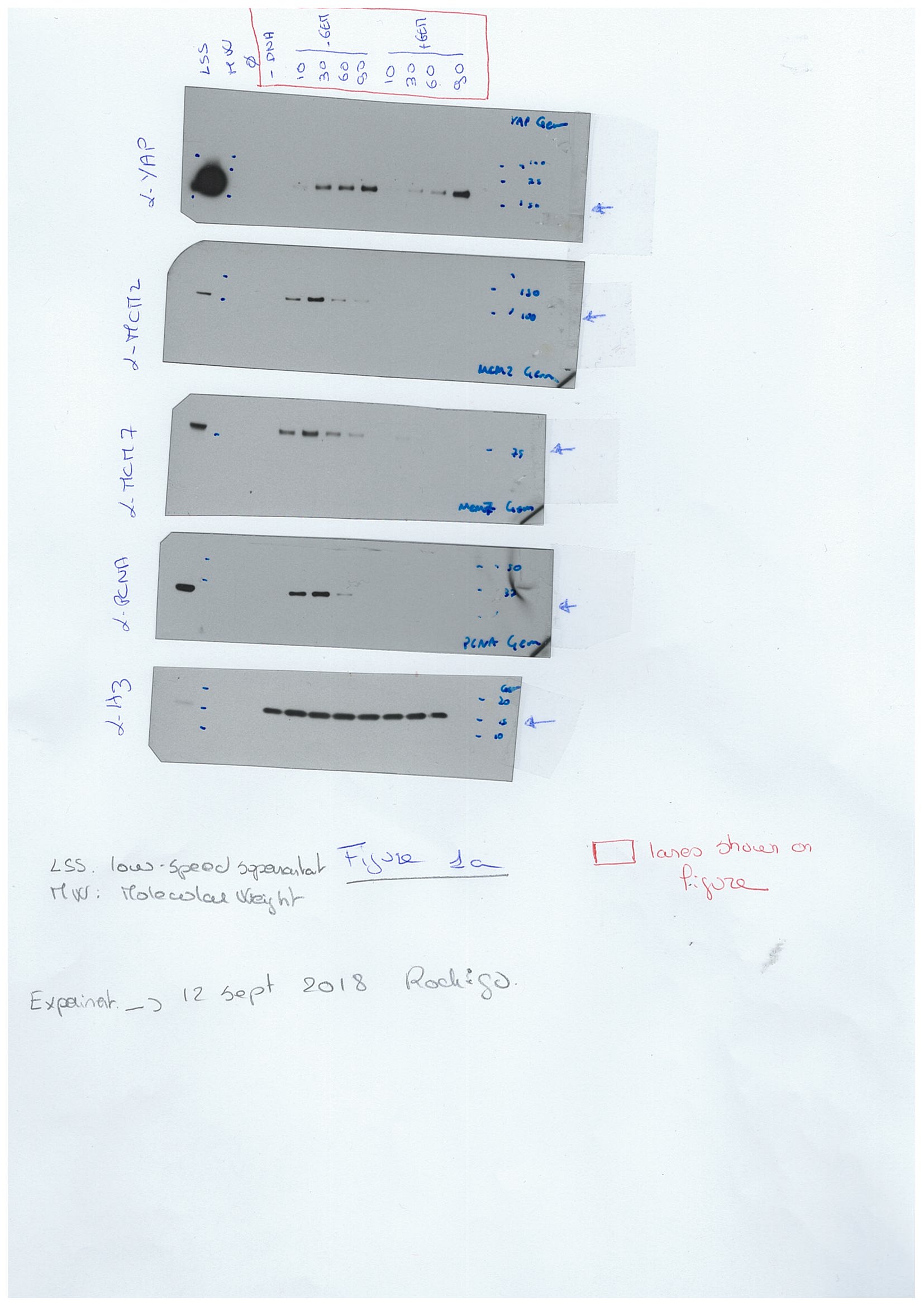

Supplement: Figure 1—source data 1. [file elife-75741-fig1-data1.zip › Figure 1-Source Data/Fig1A_OriginalBlots.JPG]

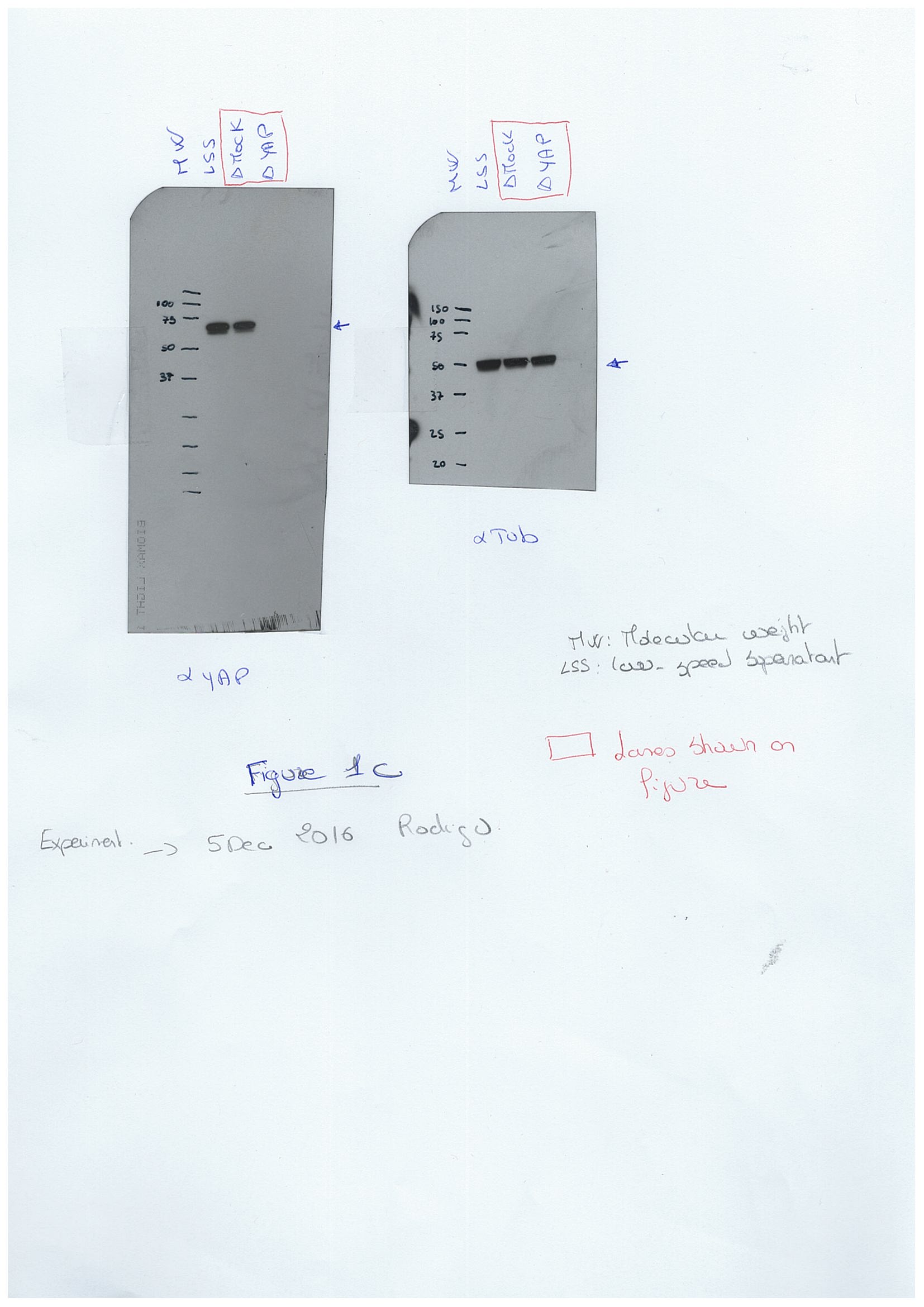

Supplement: Figure 1—source data 1. [file elife-75741-fig1-data1.zip › Figure 1-Source Data/Fig1C_OriginalBlots.JPG]

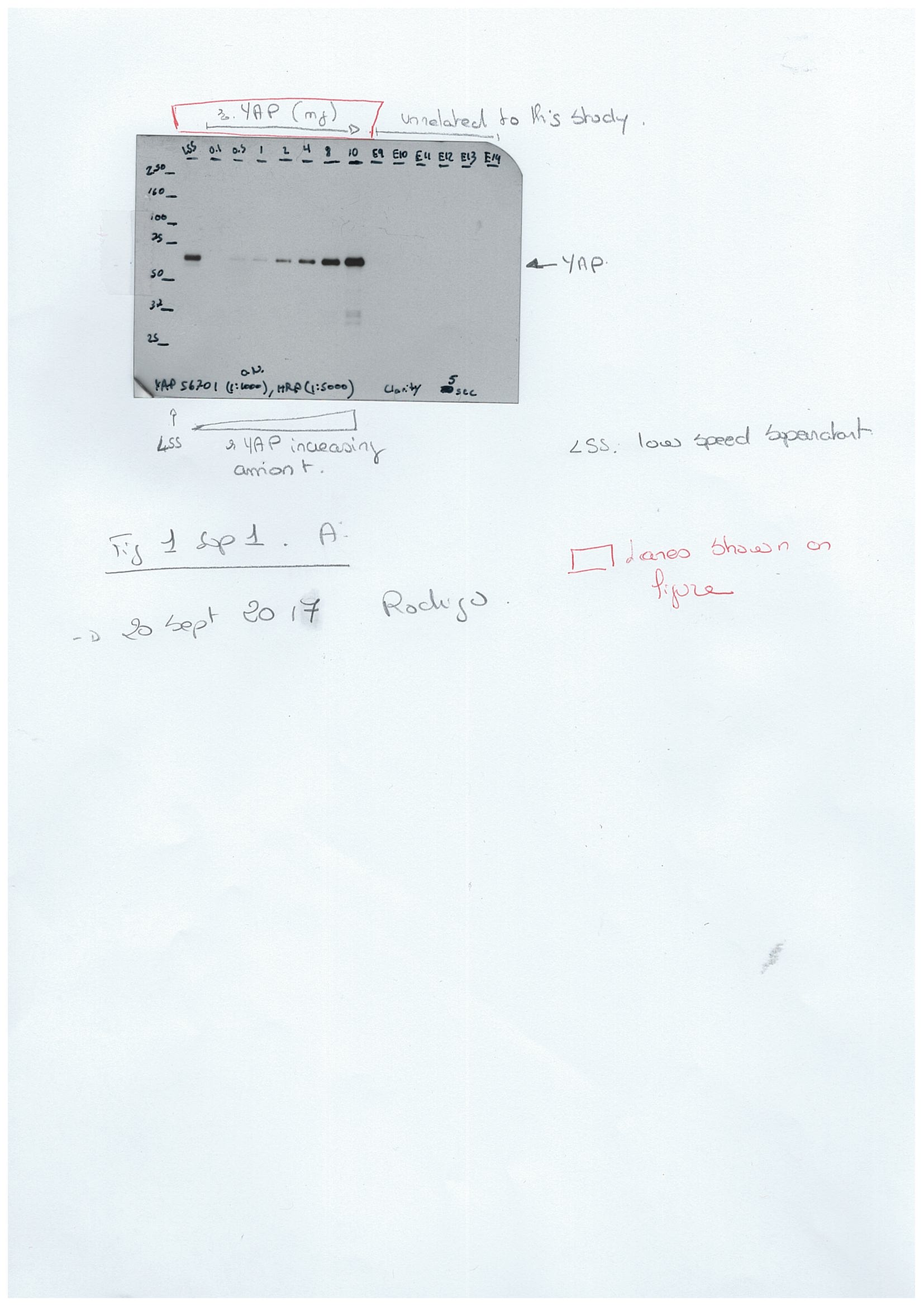

Supplement: Figure 1—figure supplement 1—source data 1. [file elife-75741-fig1-figsupp1-data1.zip › Figure 1-Figure supplement 1-Source Data/Fig1Sup1A_OriginalBlot.JPG]

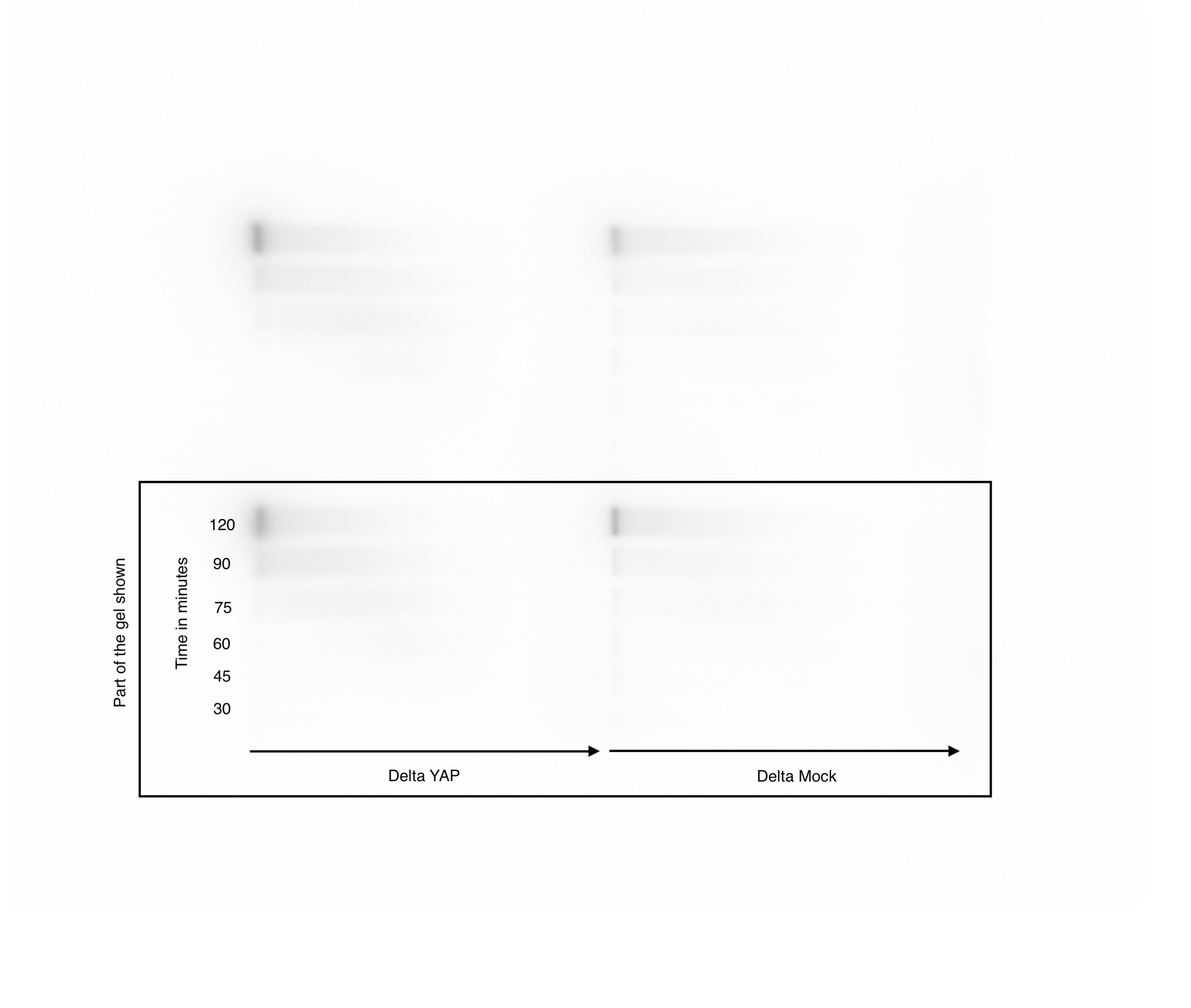

Supplement: Figure 1—figure supplement 2—source data 1. [file elife-75741-fig1-figsupp2-data1.zip › Figure 1-Figure supplement 2-Source Data/Fig1-Sup2A-YApdepletionAlkGel_annotated.tif]

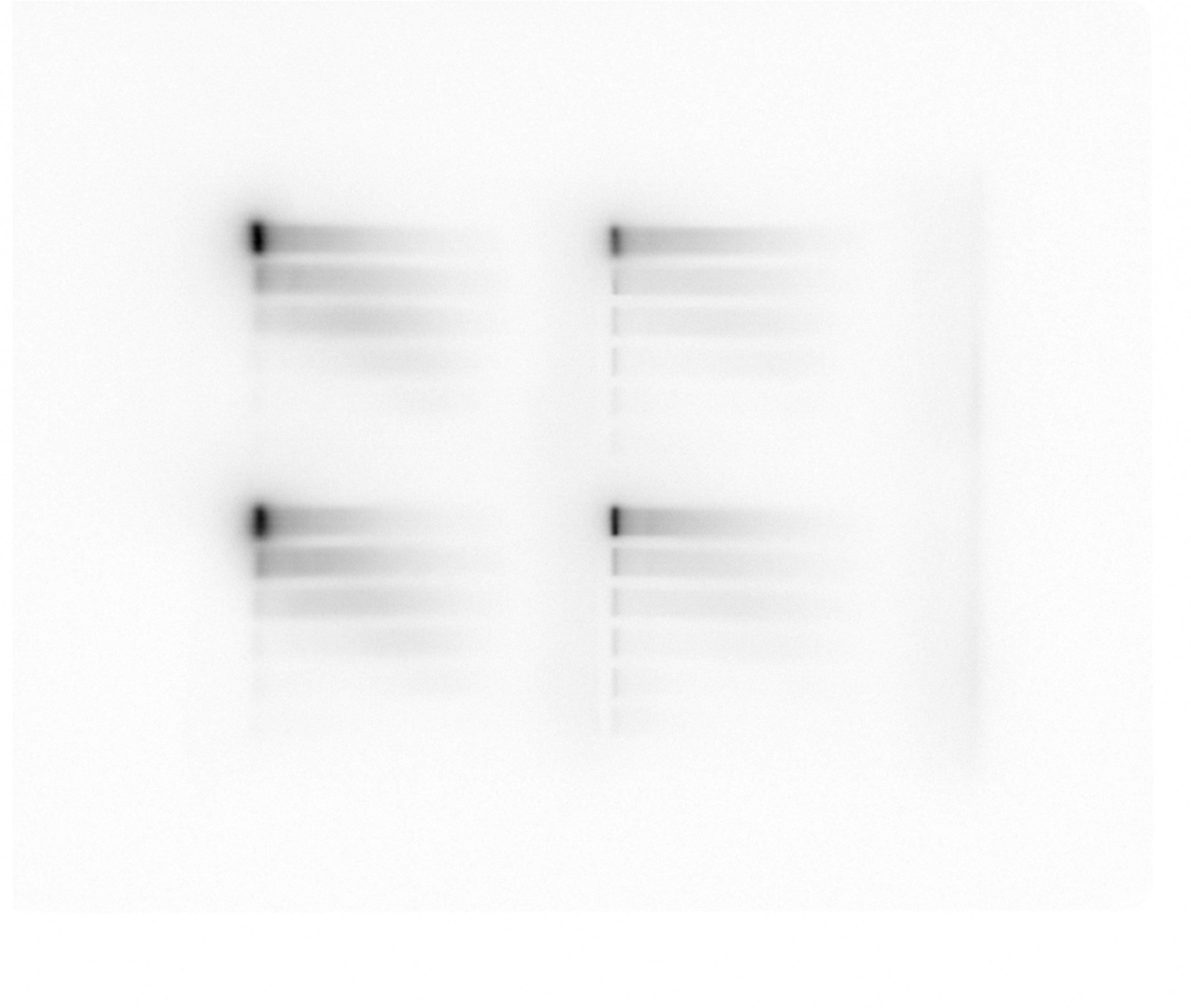

Supplement: Figure 1—figure supplement 2—source data 1. [file elife-75741-fig1-figsupp2-data1.zip › Figure 1-Figure supplement 2-Source Data/Fig1-Sup2A-YApdepletionAlkGel.tif]

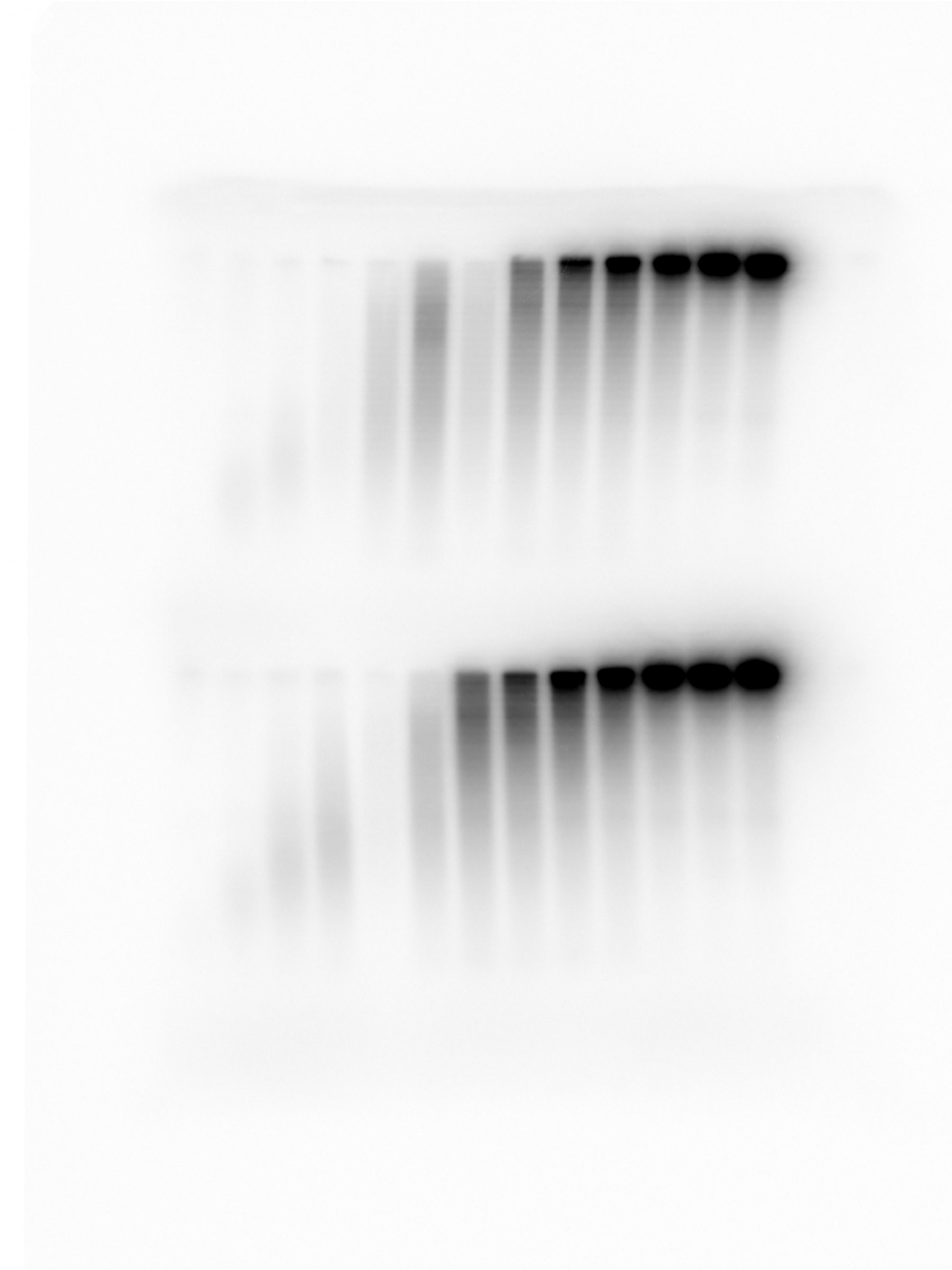

Supplement: Figure 1—figure supplement 3—source data 1. [file elife-75741-fig1-figsupp3-data1.zip › Figure 1-Figure supplement 3-Source Data/Fig1Sup3A_OriginalBlot.jpg]

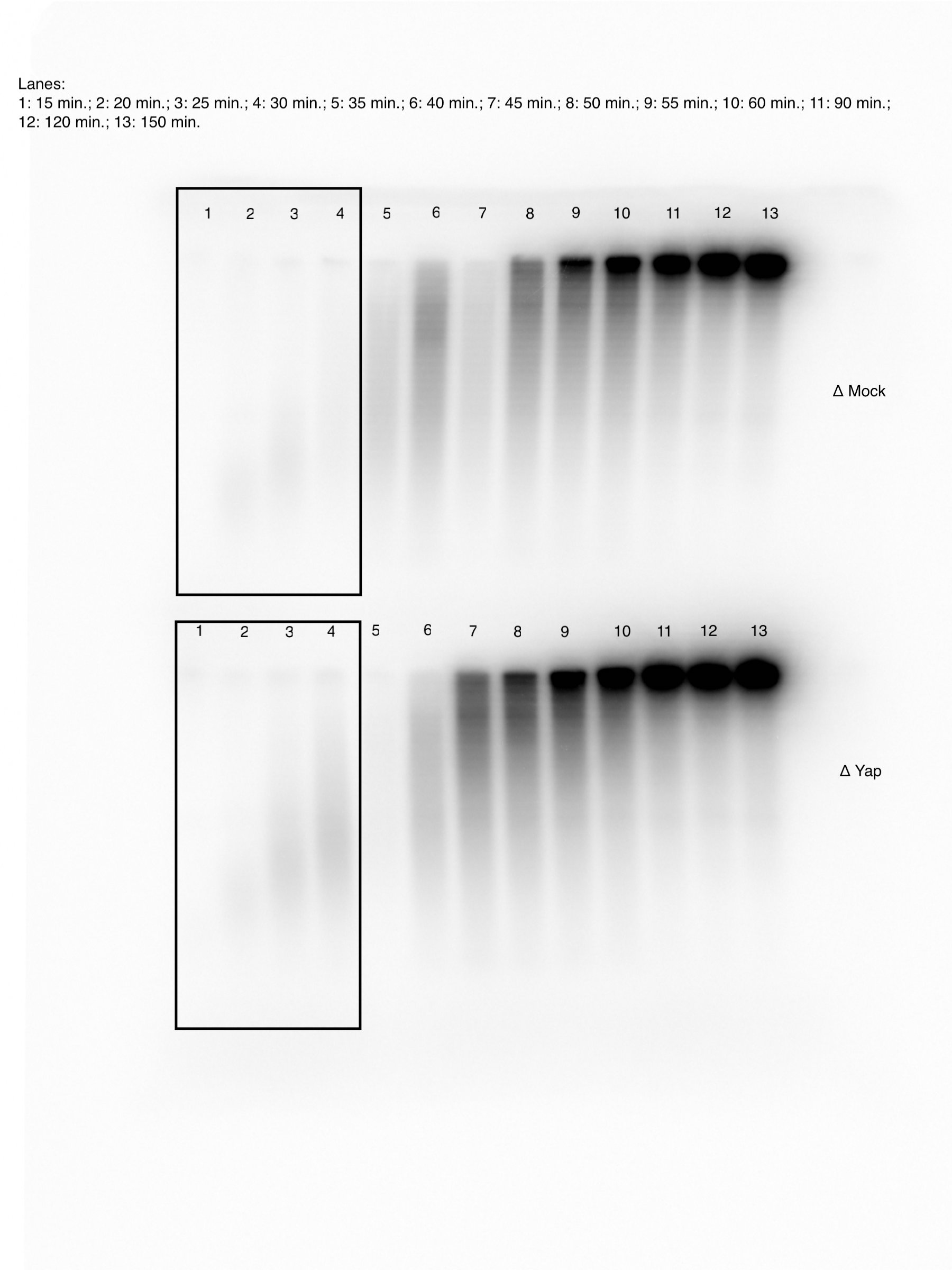

Supplement: Figure 1—figure supplement 3—source data 1. [file elife-75741-fig1-figsupp3-data1.zip › Figure 1-Figure supplement 3-Source Data/Fig1Sup3A_OriginalBlot_annotated.jpg]

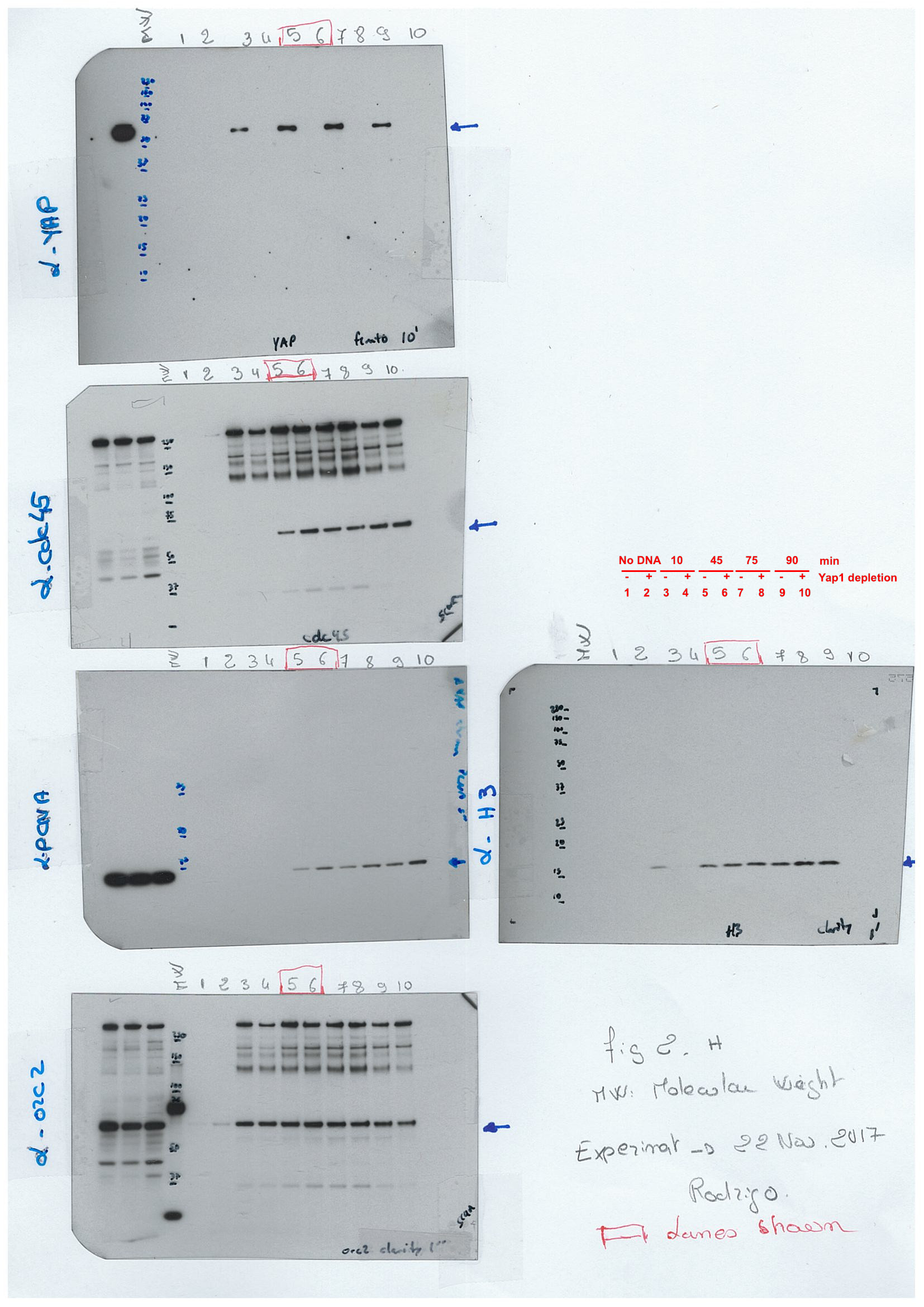

Supplement: Figure 2—source data 1. [file elife-75741-fig2-data1.zip › Figure 2-Source Data/Fig2H_OriginalBlots.JPG]

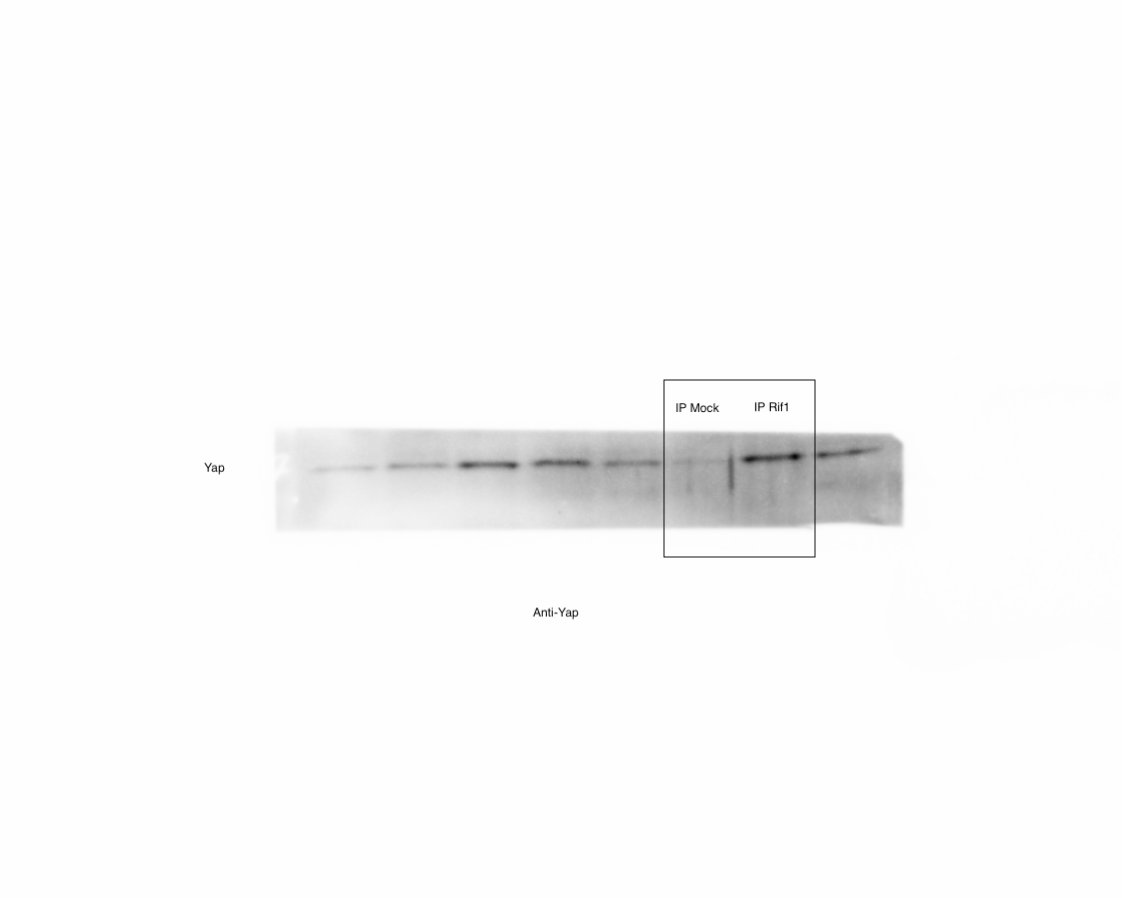

Supplement: Figure 3—source data 1. [file elife-75741-fig3-data1.zip › Figure 3-Source Data/Fig3B_Rightpannel_antiYAP_OriginalBlot_annotated.jpg]

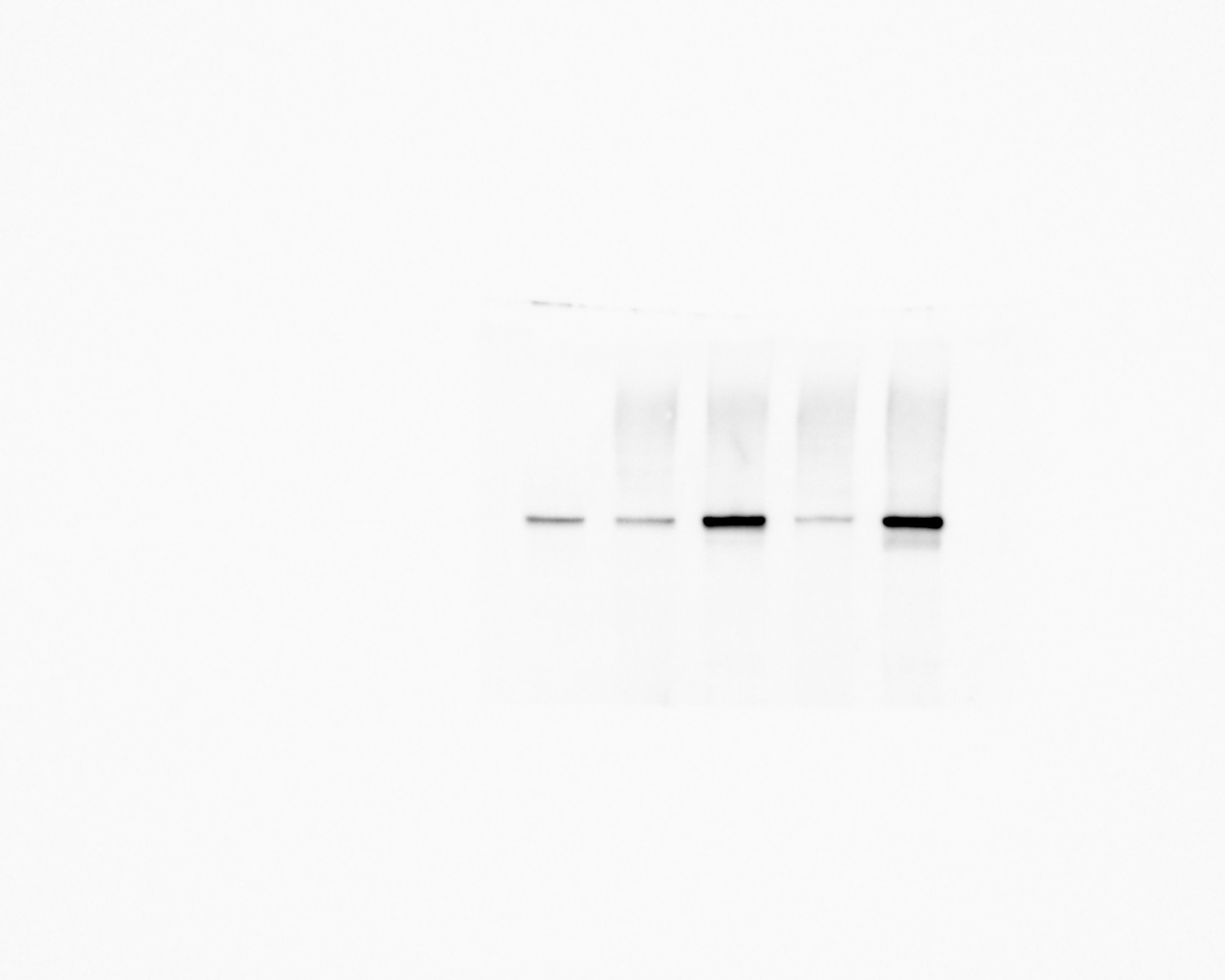

Supplement: Figure 3—source data 1. [file elife-75741-fig3-data1.zip › Figure 3-Source Data/Fig3B_Rightpannel_antiRIF_OriginalBlot.jpg]

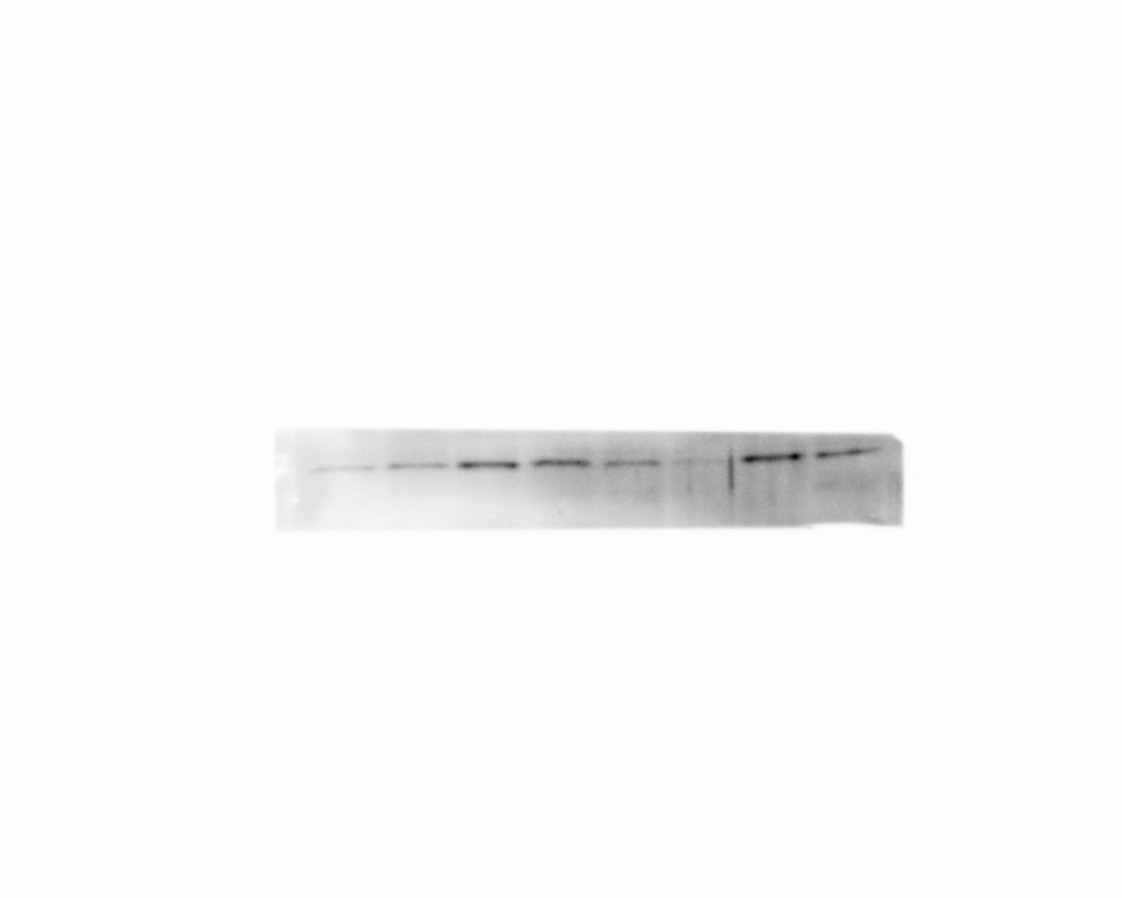

Supplement: Figure 3—source data 1. [file elife-75741-fig3-data1.zip › Figure 3-Source Data/Fig3B_Rightpannel_antiYAP_OriginalBlot.jpg]

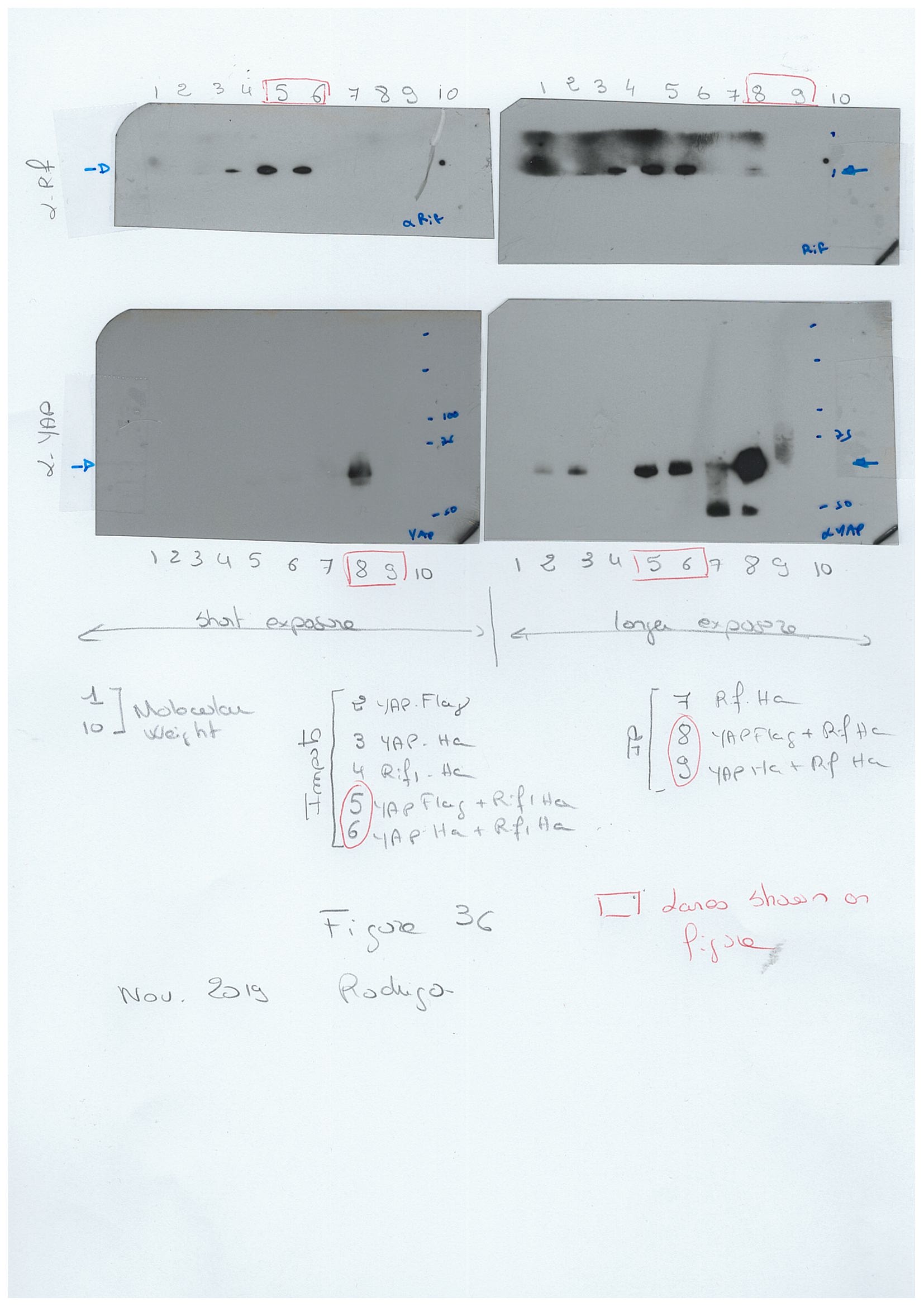

Supplement: Figure 3—source data 1. [file elife-75741-fig3-data1.zip › Figure 3-Source Data/Fig3C_Originalblots.JPG]

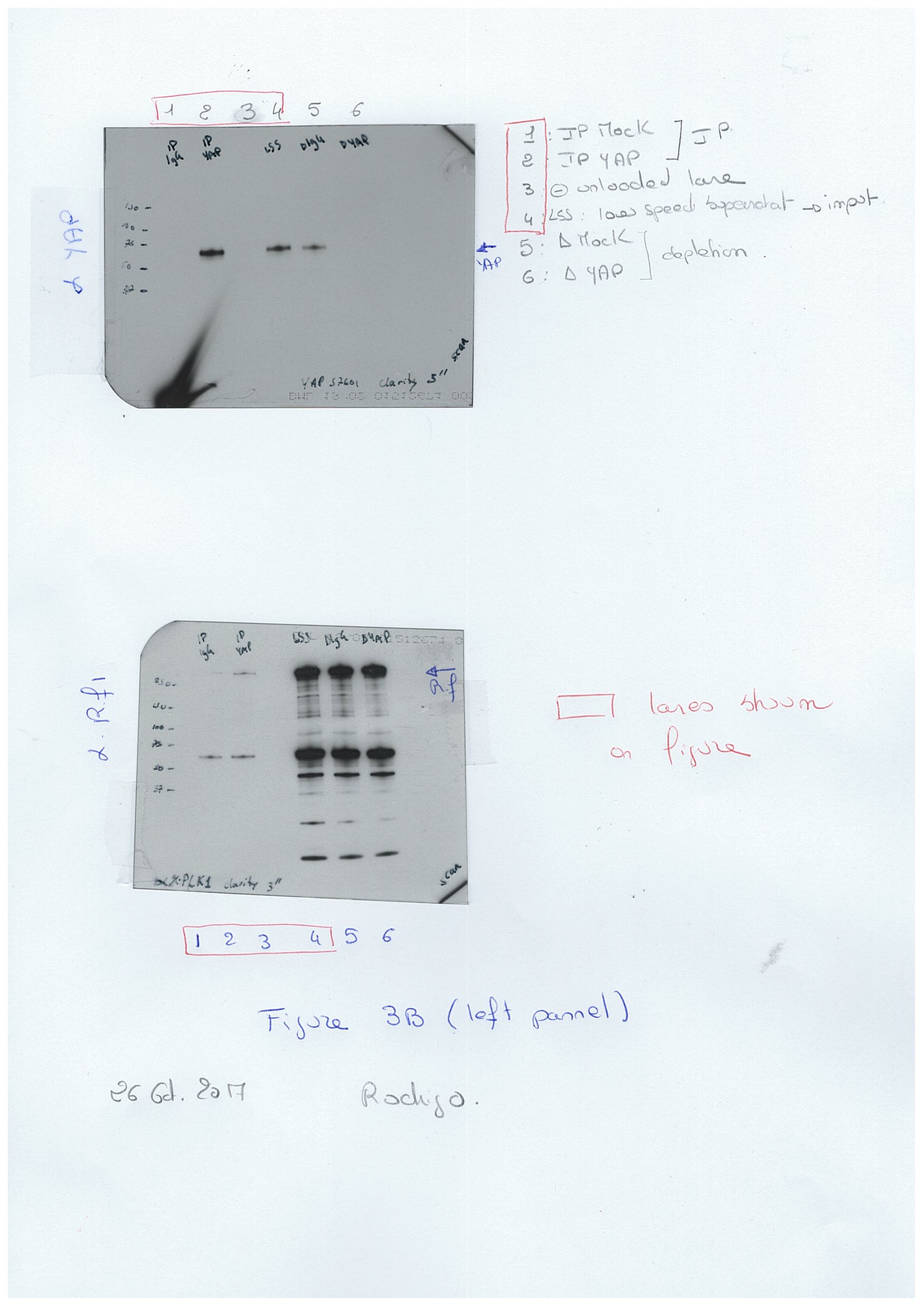

Supplement: Figure 3—source data 1. [file elife-75741-fig3-data1.zip › Figure 3-Source Data/Fig3B_Leftpannel_originalblots.JPG]

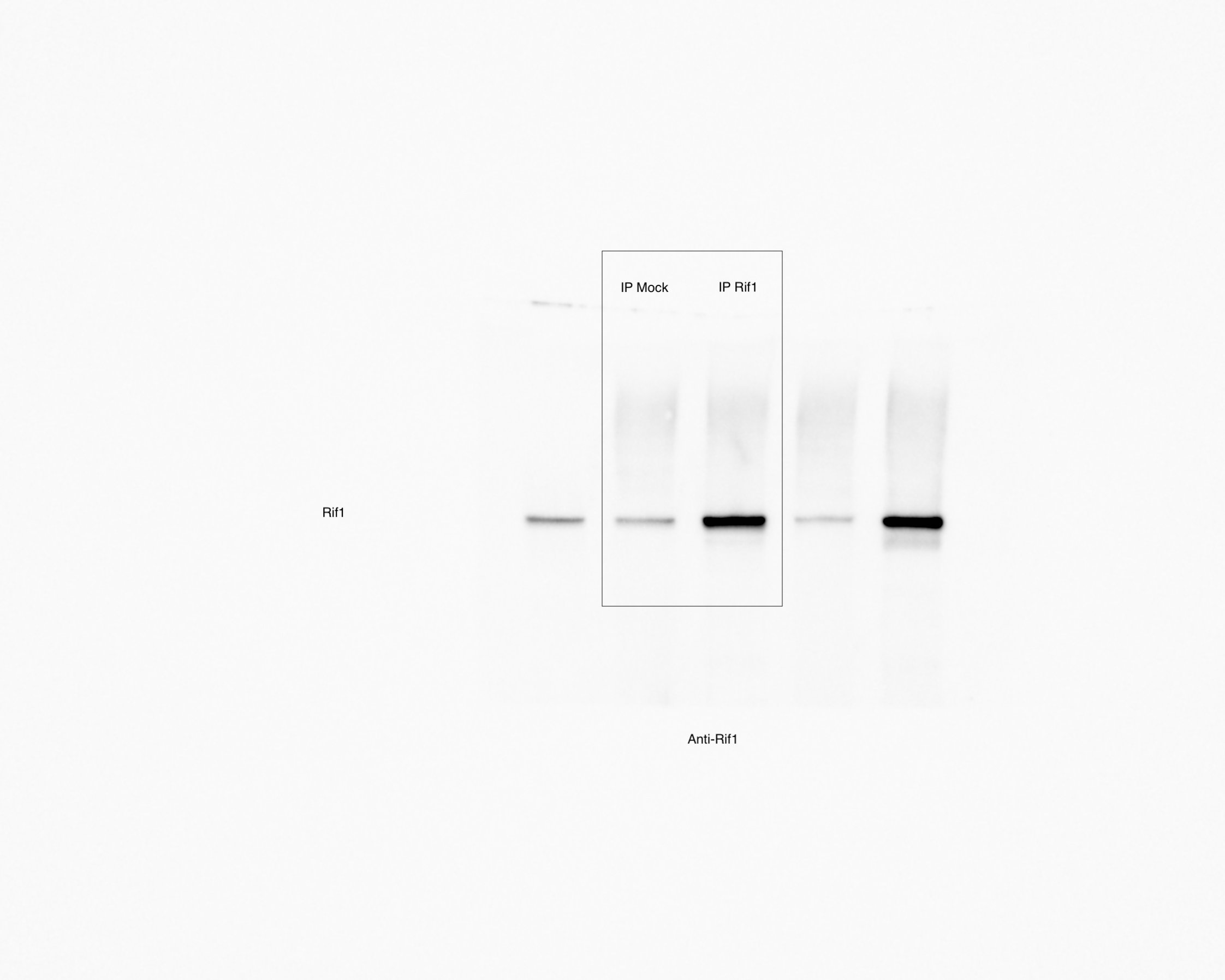

Supplement: Figure 3—source data 1. [file elife-75741-fig3-data1.zip › Figure 3-Source Data/Fig3B_Rightpannel_antiRIF_OriginalBlot_annotated.jpg]

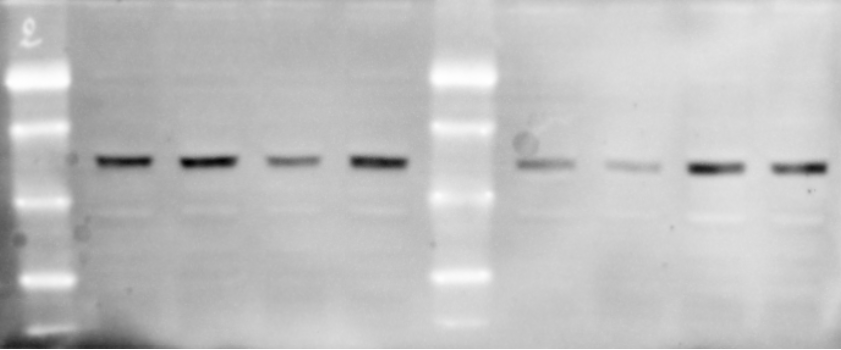

Supplement: Figure 4—source data 1. [file elife-75741-fig4-data1.zip › Figure 4-Source Data/Fig4A_WBlotRif1depletionLSS/Tub.tif]

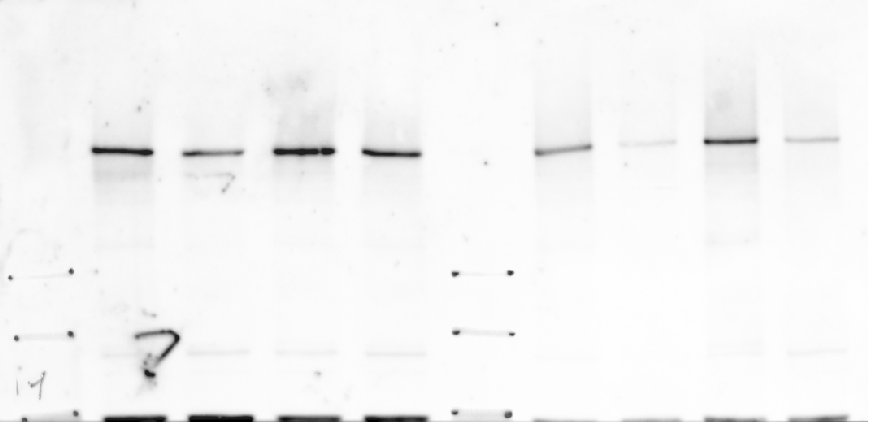

Supplement: Figure 4—source data 1. [file elife-75741-fig4-data1.zip › Figure 4-Source Data/Fig4A_WBlotRif1depletionLSS/Color+Rif1.tif]

## Slide 1
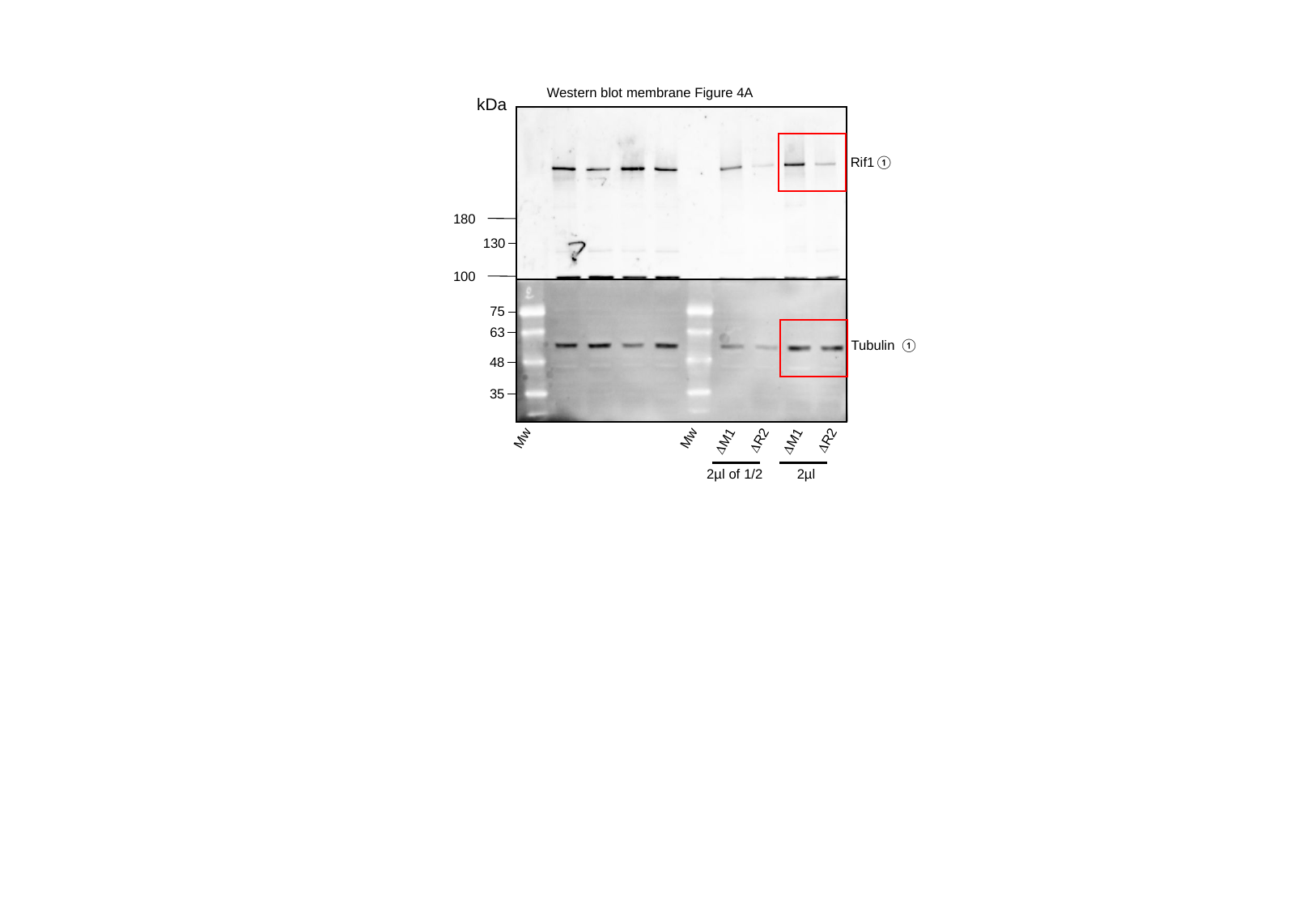

Western blot membrane Figure 4A
kDa
Rif1
①
180
130
100
75
63
①
Tubulin
48
35
Mw
Mw
ΔR2
ΔR2
ΔM1
ΔM1
2µl of 1/2
2µl

Supplement: Figure 4—source data 1. [file elife-75741-fig4-data1.zip › Figure 4-Source Data/Fig4A_WBlotRif1depletionLSS/Figure4AWBRif1+Tubulin.pptx]

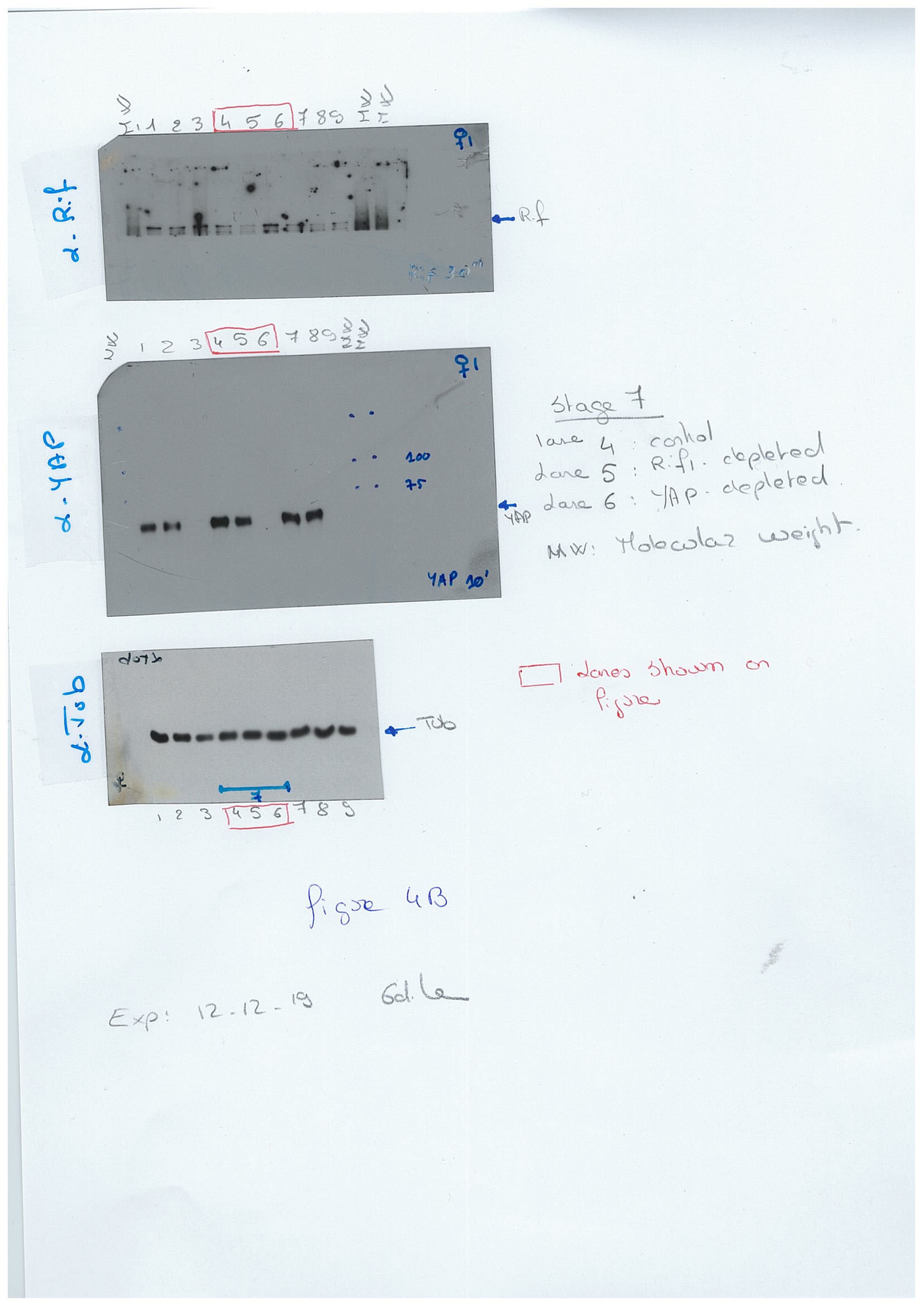

Supplement: Figure 5—source data 1. [file elife-75741-fig5-data1.zip › Figure 5-Source Data/Fig5D_Originalblots.JPG]

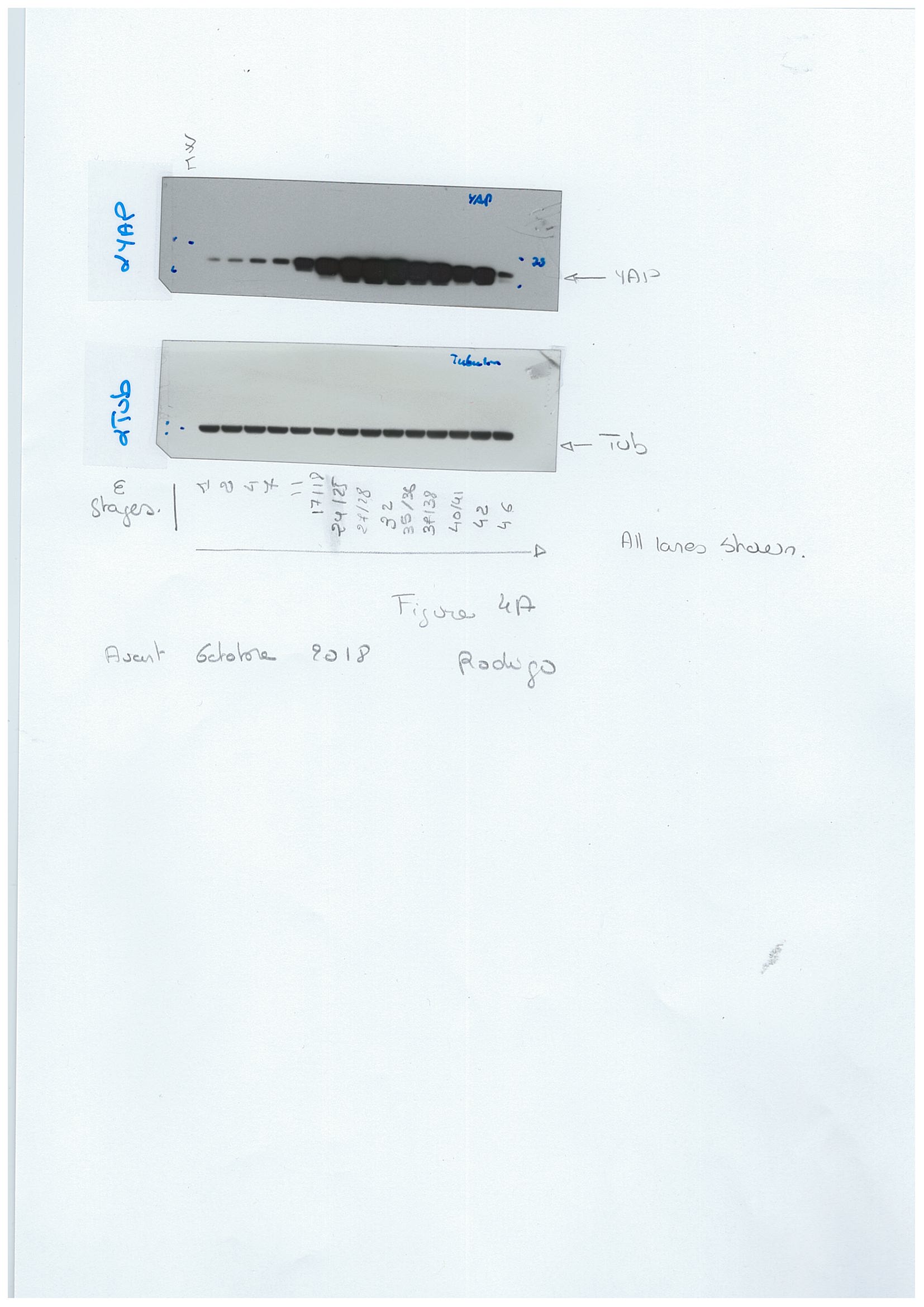

Supplement: Figure 5—source data 1. [file elife-75741-fig5-data1.zip › Figure 5-Source Data/Fig5A_OriginalBlots.JPG]

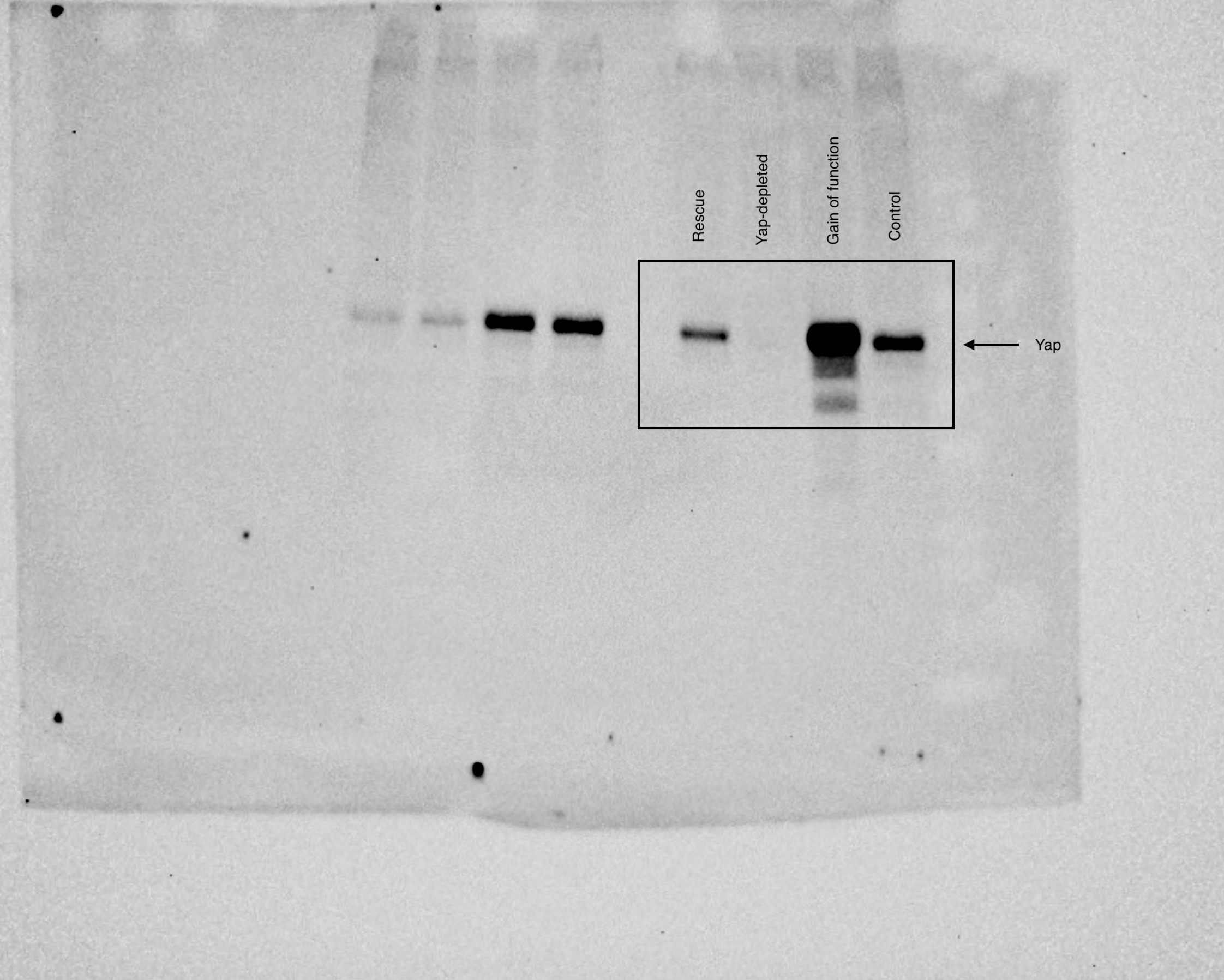

Supplement: Figure 5—source data 1. [file elife-75741-fig5-data1.zip › Figure 5-Source Data/Fig5C_OriginalBlots_Yap_Annotated.tif]

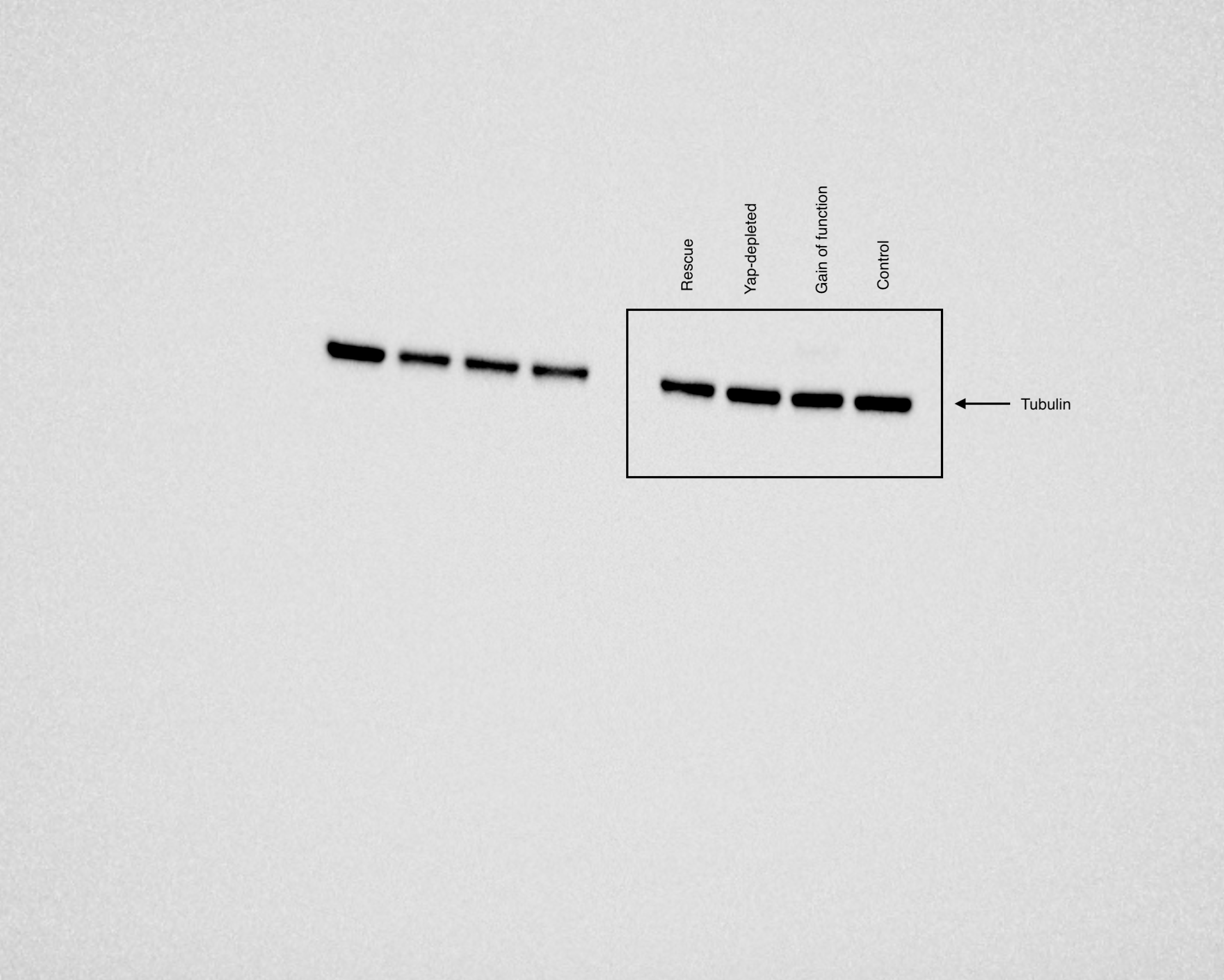

Supplement: Figure 5—source data 1. [file elife-75741-fig5-data1.zip › Figure 5-Source Data/Fig5C_OriginalBlots_Tub_Annotated.tif]

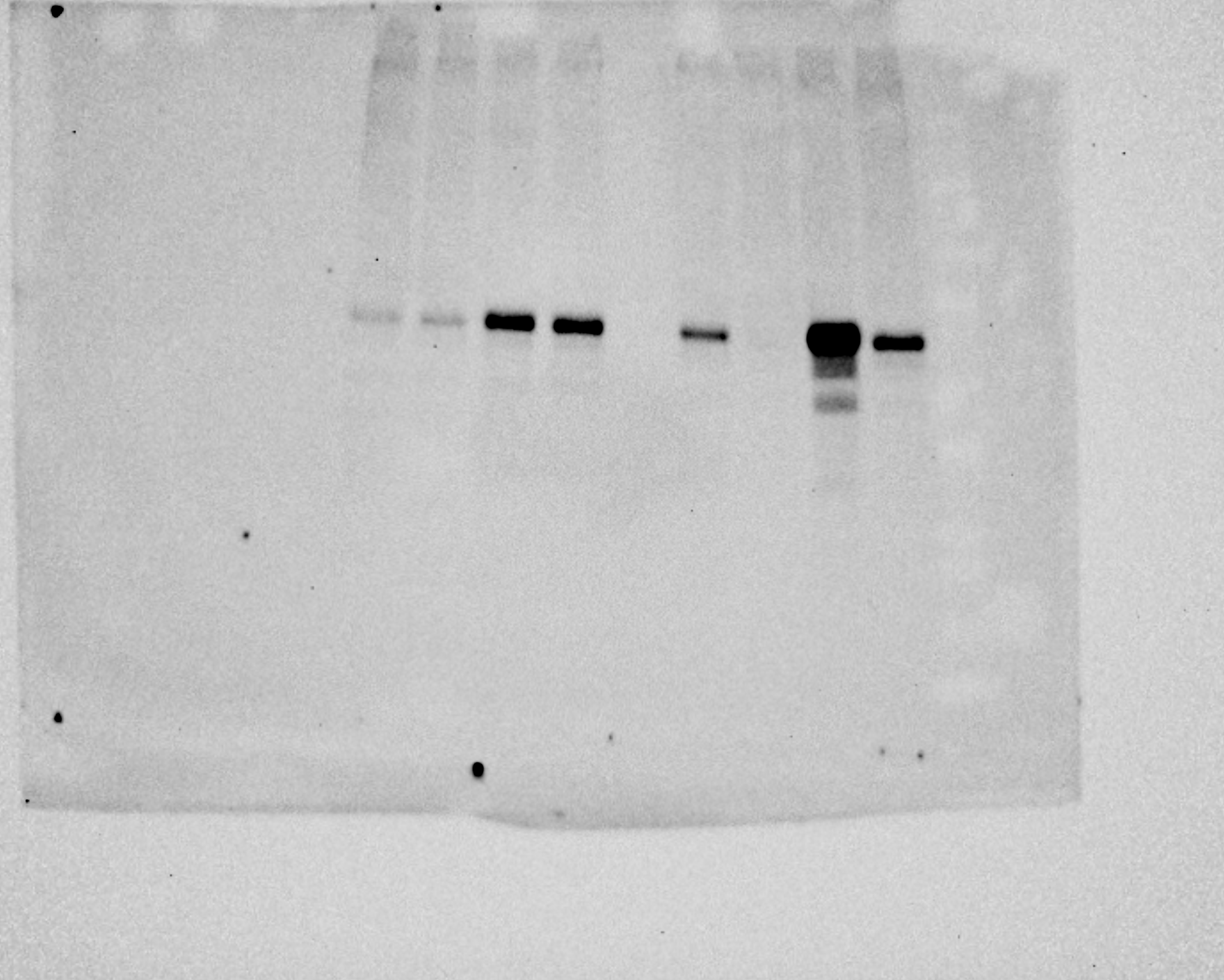

Supplement: Figure 5—source data 1. [file elife-75741-fig5-data1.zip › Figure 5-Source Data/Fig5C_OriginalBlots_Yap.tif]

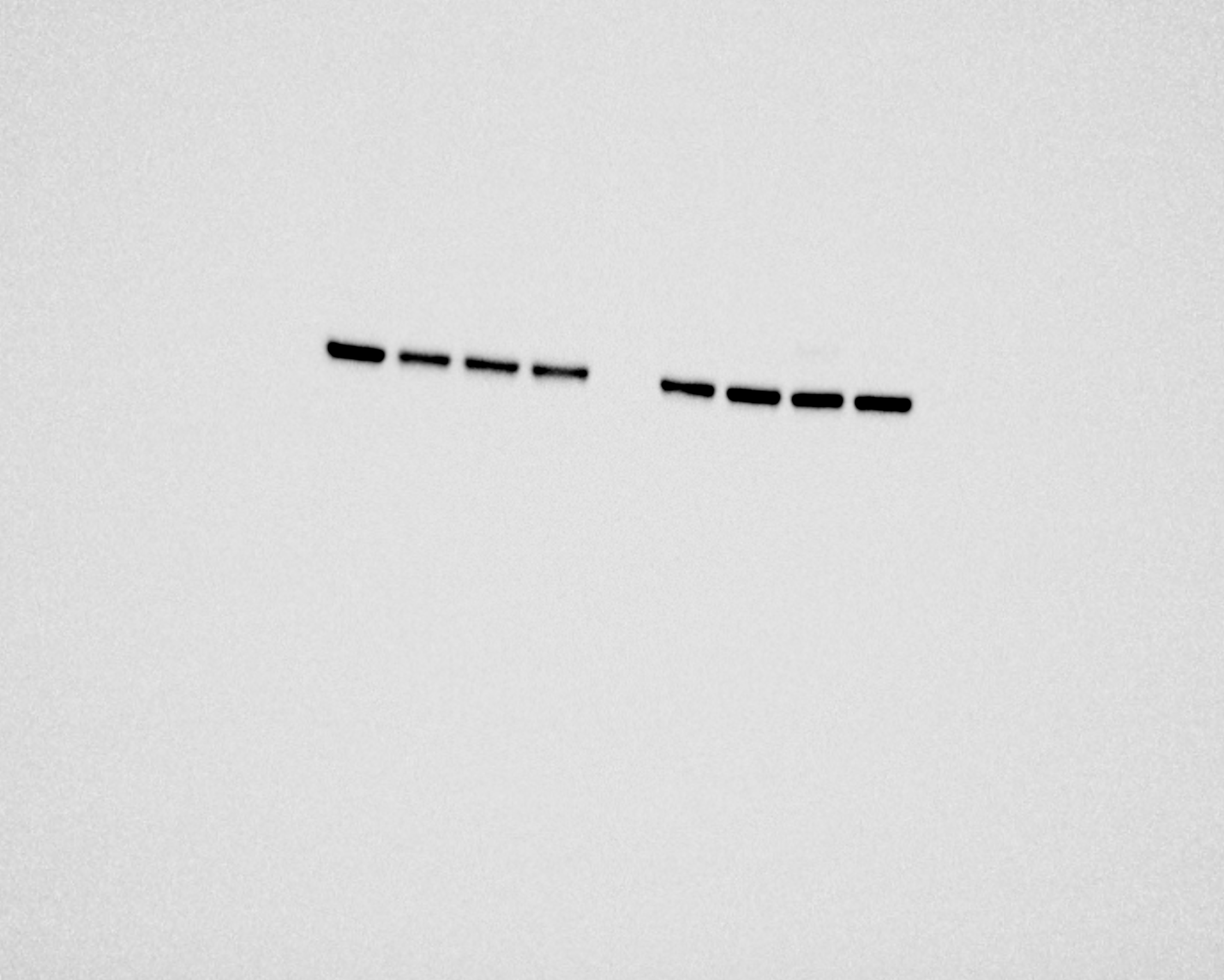

Supplement: Figure 5—source data 1. [file elife-75741-fig5-data1.zip › Figure 5-Source Data/Fig5C_OriginalBlots_Tub.tif]

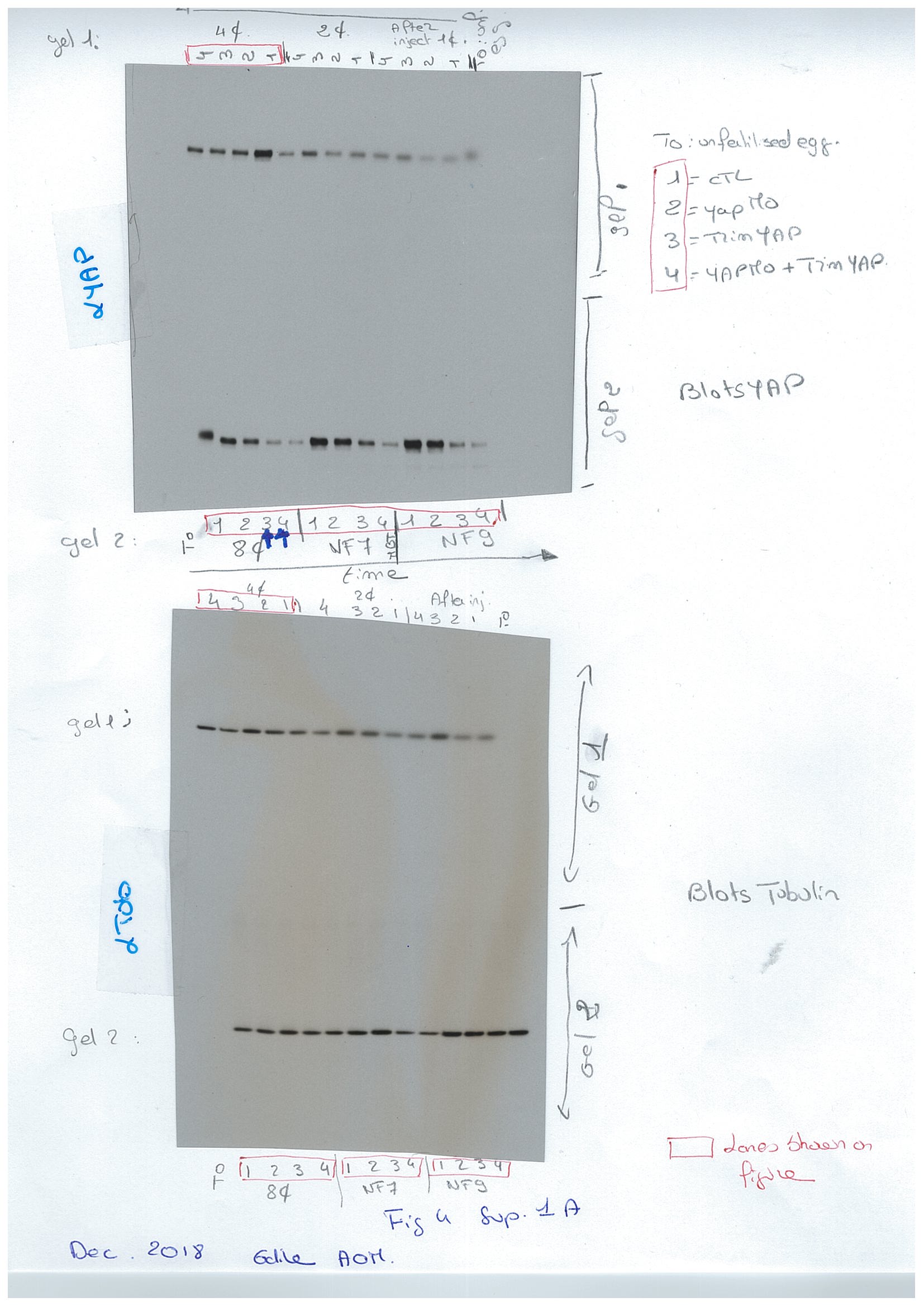

Supplement: Figure 5—figure supplement 1—source data 1. [file elife-75741-fig5-figsupp1-data1.zip › Figure 5-Figure suppement 1-Source Data/Fig5Sup1A_OriginalBlots.JPG]

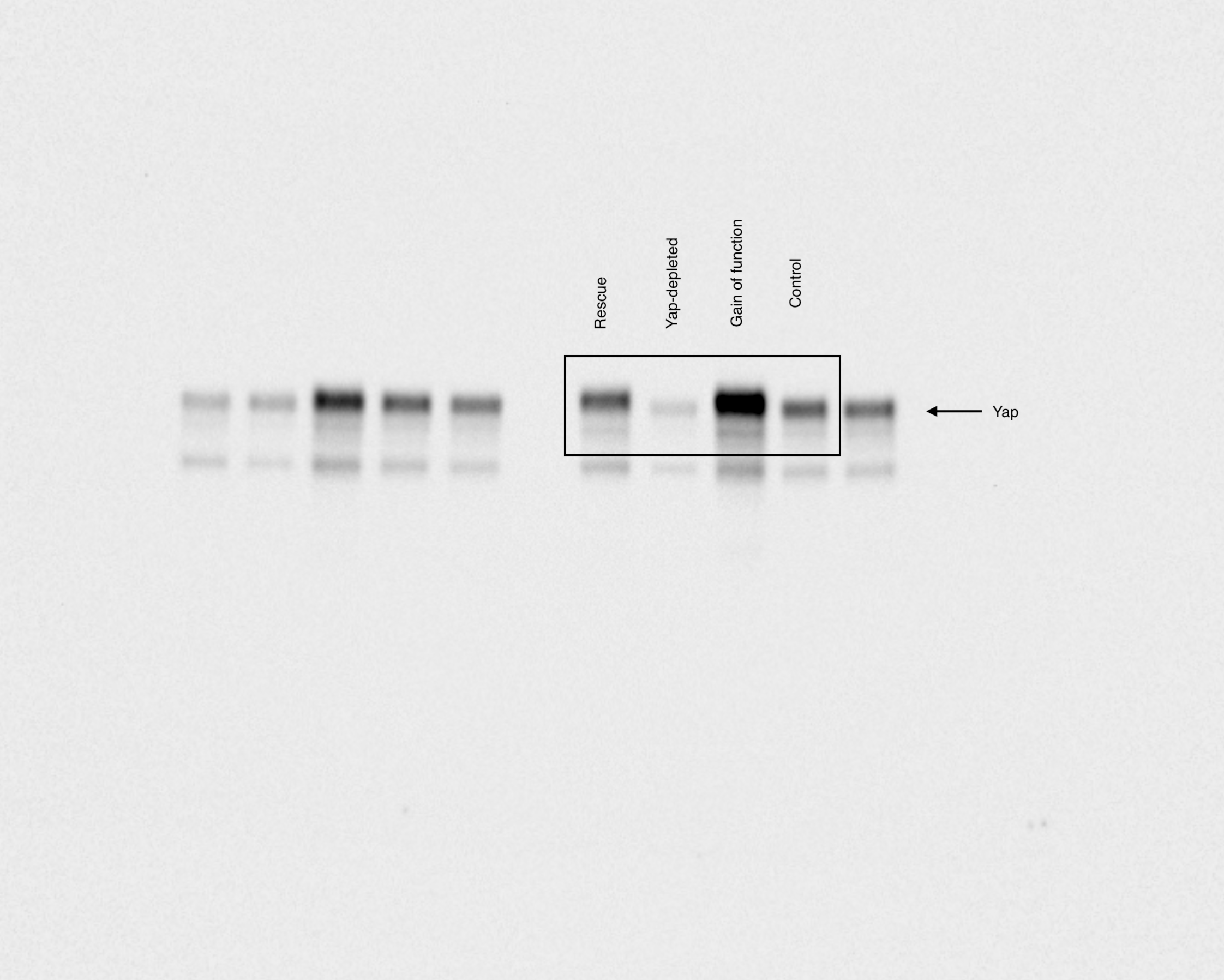

Supplement: Figure 5—figure supplement 1—source data 1. [file elife-75741-fig5-figsupp1-data1.zip › Figure 5-Figure suppement 1-Source Data/Fig5Sup1B-OriginalBlots_Yap_annotated.tif]

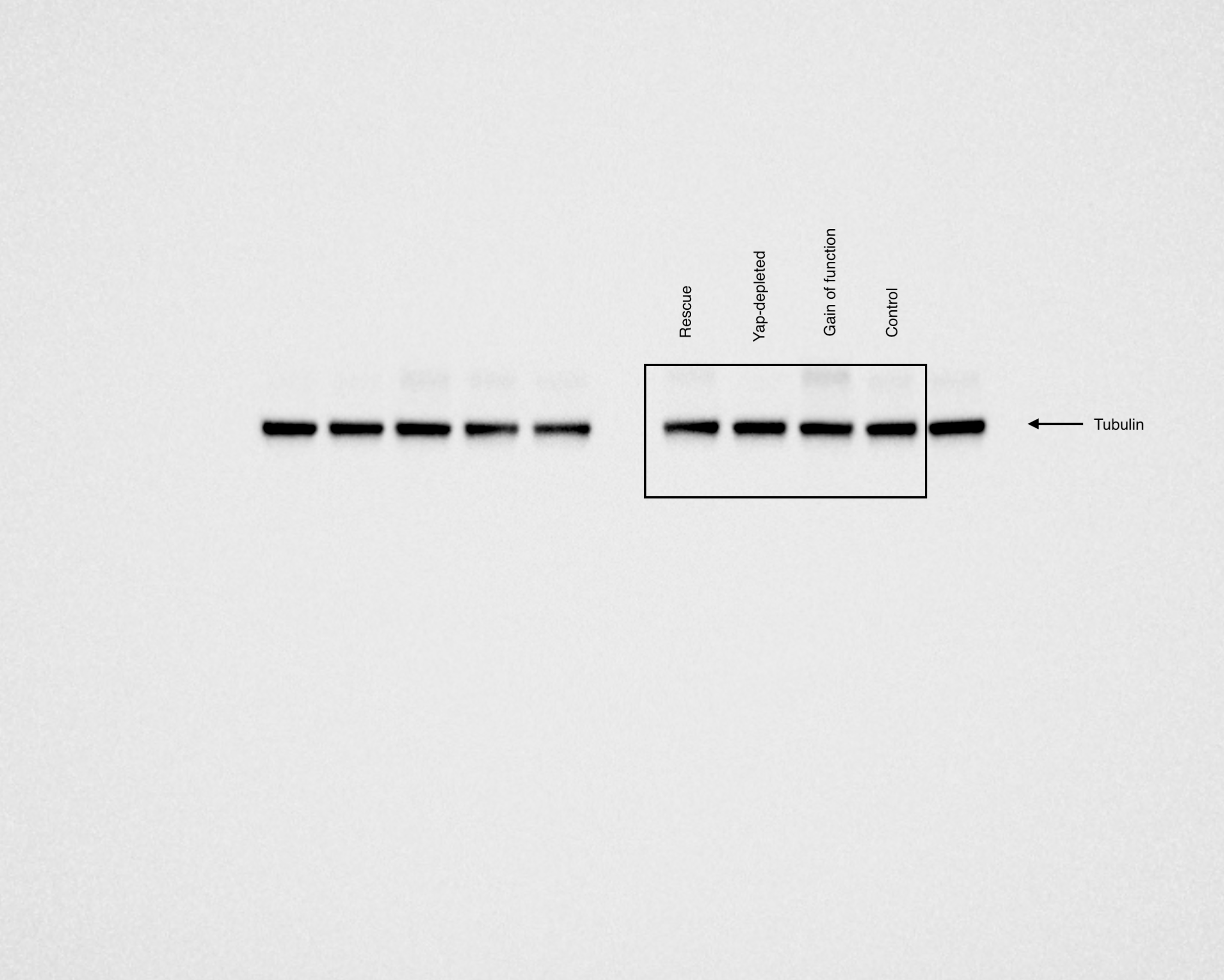

Supplement: Figure 5—figure supplement 1—source data 1. [file elife-75741-fig5-figsupp1-data1.zip › Figure 5-Figure suppement 1-Source Data/Fig5Sup1B-OriginalBlots_Tub_annotated.tif]

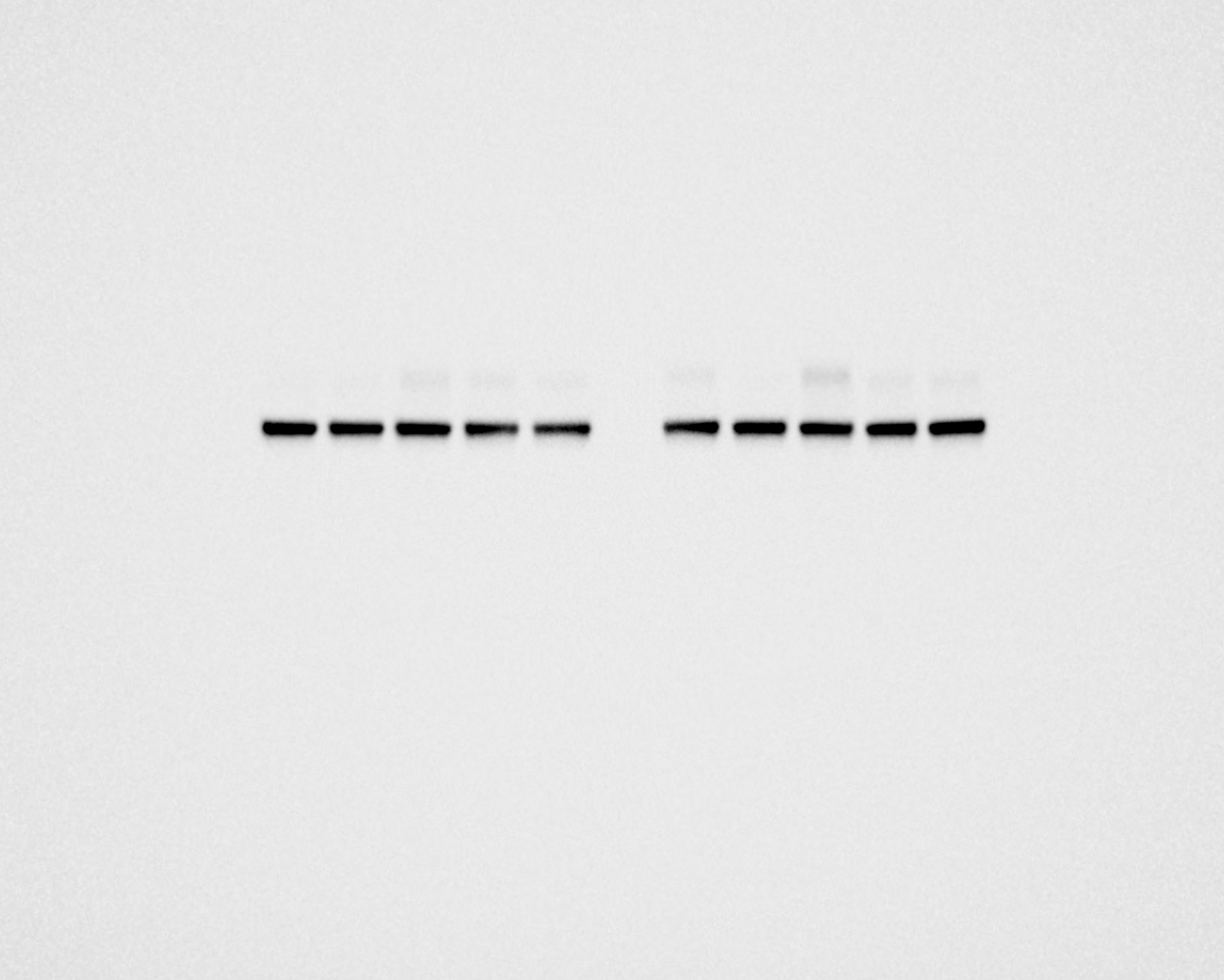

Supplement: Figure 5—figure supplement 1—source data 1. [file elife-75741-fig5-figsupp1-data1.zip › Figure 5-Figure suppement 1-Source Data/Fig5Sup1B-OriginalBlots_Tub.tif]

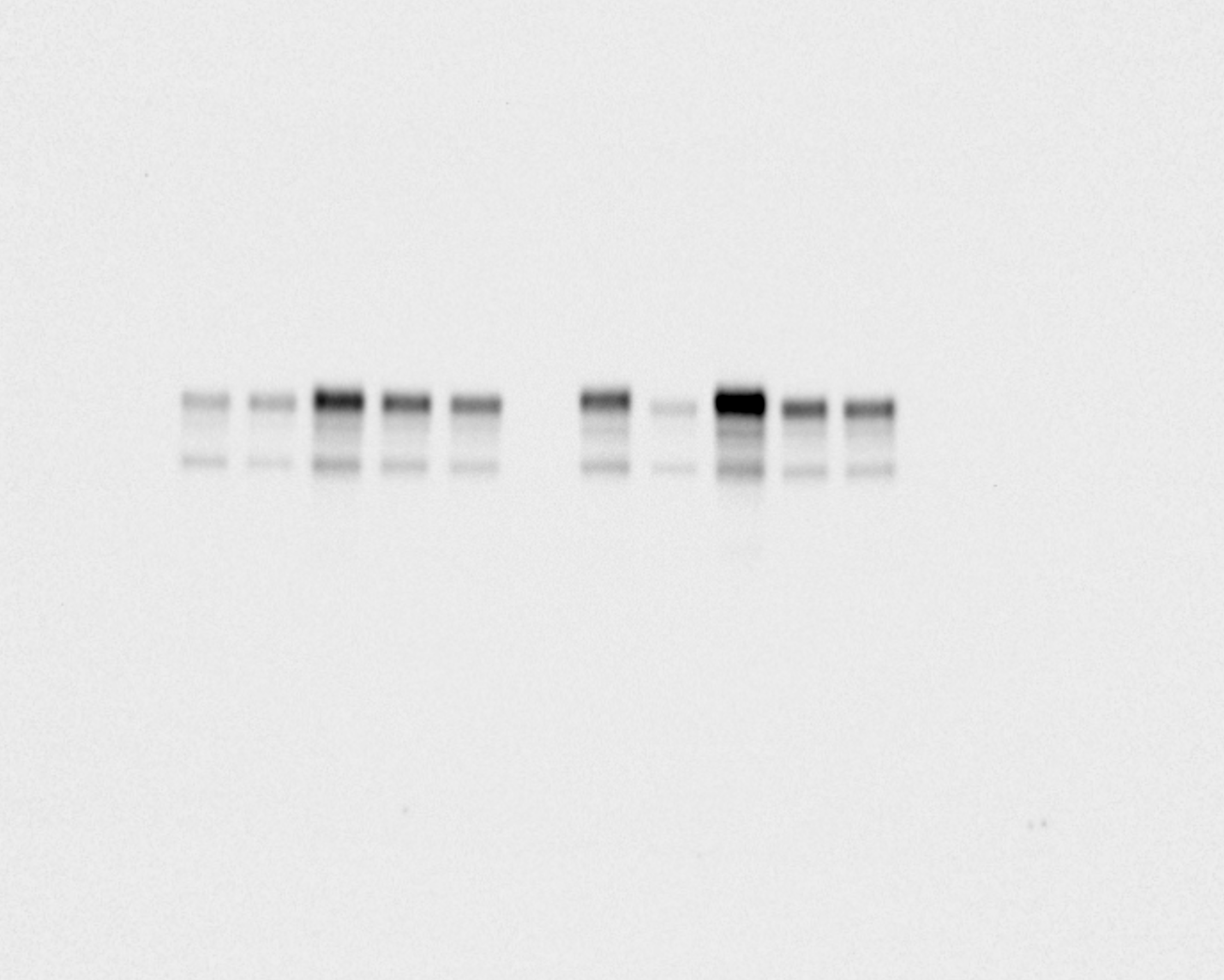

Supplement: Figure 5—figure supplement 1—source data 1. [file elife-75741-fig5-figsupp1-data1.zip › Figure 5-Figure suppement 1-Source Data/Fig5Sup1B-OriginalBlots_Yap.tif]

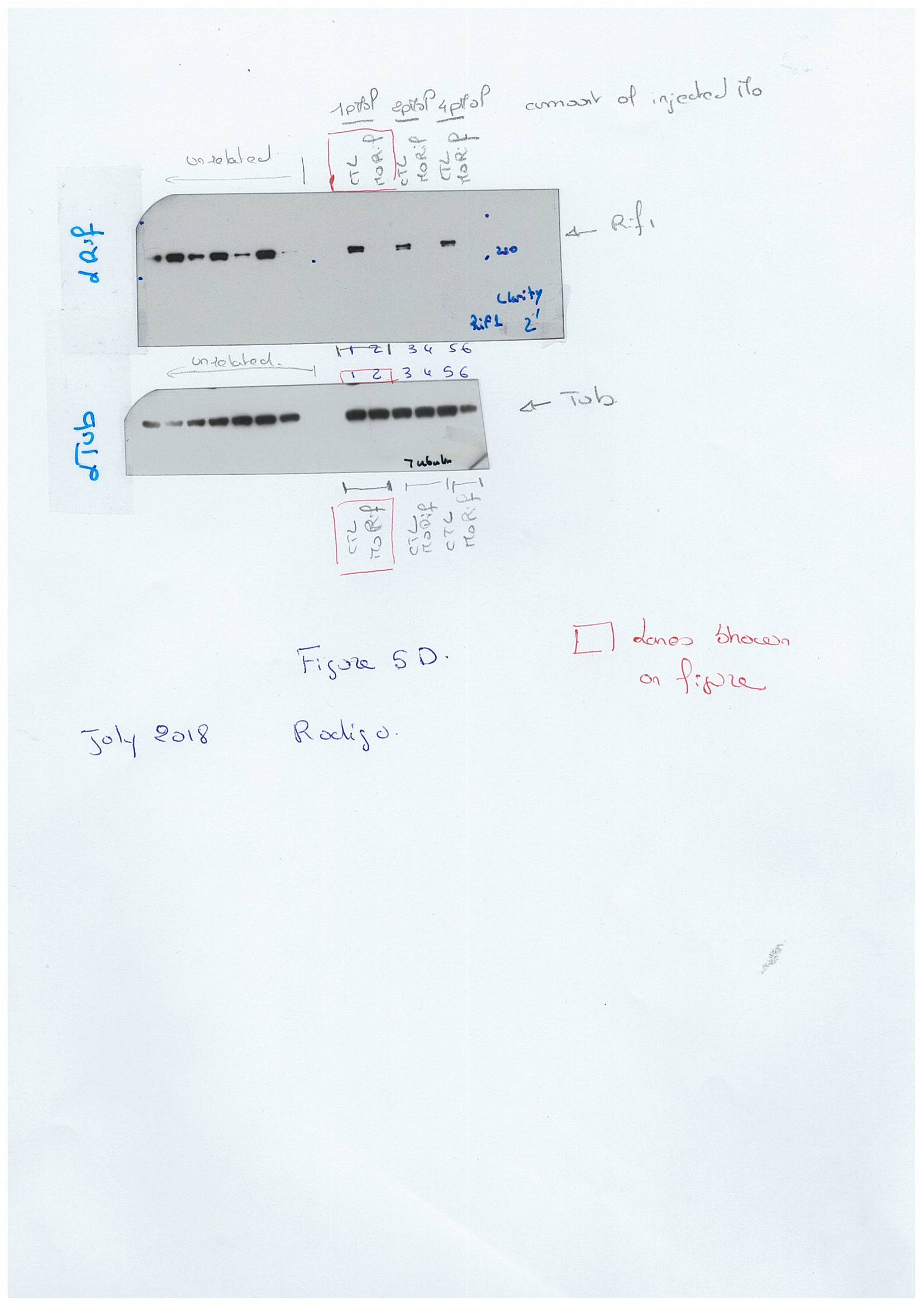

Supplement: Figure 6—source data 1. [file elife-75741-fig6-data1.zip › Figure 6-Source Data/Fig6D_OriginalBlots.JPG]

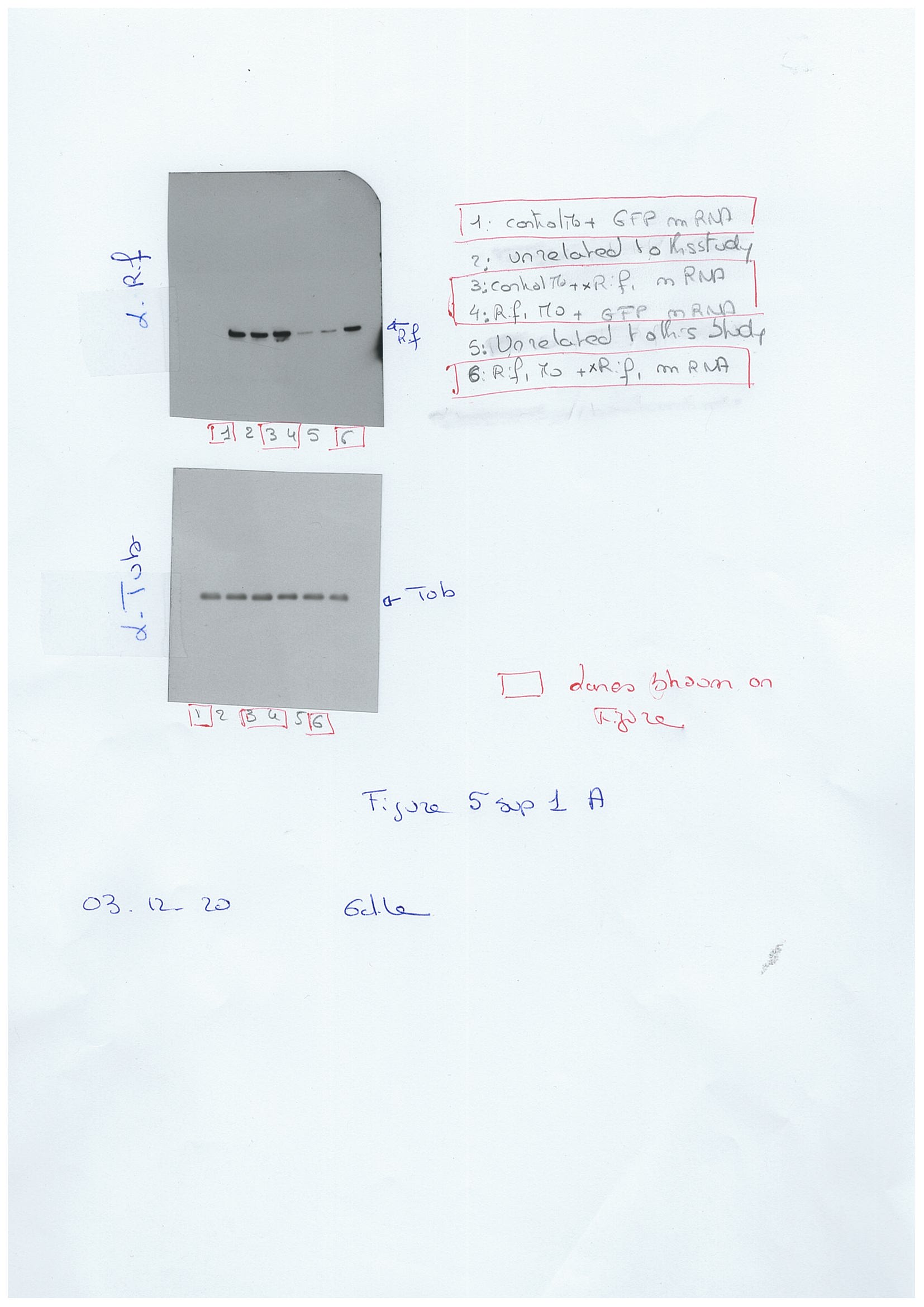

Supplement: Figure 6—figure supplement 2—source data 1. [file elife-75741-fig6-figsupp2-data1.zip › Figure 6-Figure supplement 2-Source Data/Fig6Sup2A_OrignalBlots.JPG]
