# Supplementary material for: A non-transcriptional function of Yap regulates the DNA replication program in Xenopus laevis
Source: eLife. 2022 Jul 15;11:e75741. doi: 10.7554/eLife.75741 (PMC9328763; doi:10.7554/eLife.75741)
Supplement: Supplementary file 1. — Extended DNA combing data to Figure 2 of 2 independent experiments, Replicate 1 and Replicate 2. [file elife-75741-supp1.docx]

**Supplementary File 1**

**Depletion of Yap increases replication origin firing in *Xenopus* egg extracts**

Extended DNA combing data to Figure 2

2 independent experiments, Replicate 1 and Replicate 2

| Replicate | **Replicate 1** | | | | **Replicate 2** | | | |
| --- | --- | --- | --- | --- | --- | --- | --- | --- |
| time | **35 min** |  | **55 min** |  | **65 min** |  | **80 min** |  |
| condition | **∆Mock** | **∆Yap** | **∆Mock** | **∆Yap** | **∆Mock** | **∆Yap** | **∆Mock** | **∆Yap** |
| Analysed DNA (kb) | 4653.09 | 3773.9 | 11332.1 | 12784.59 | 15674.9 | 14752,0 | 18163.52 | 17709.8 |
| Replicated DNA(kb) | 92.95 | 131.04 | 1674.66 | 5688.02 | 883.5 | 2498.88 | 1909.44 | 4003.2 |
| **Replicated fraction** | **0.02** | **0.035** | **0.15** | **0.45** | **0.056** | **0.169** | **0.105** | **0.226** |
| **∆Yap/Mock replicated fraction** | **1.75** | | **3.0** | | **3.0** | | **2.1** | |
| Number of analysed fibers | 91 | 94 | 197 | 214 | 145 | 197 | 221 | 192 |
| Number of fully replicated fibers | 0 | 0 | 3 | 25 | 0 | 1 | 1 | 5 |
| Number of unreplicated fibers | 66 | 63 | 111 | 46 | 50 | 40 | 78 | 45 |
| **% of unreplicated fibers** | **72.5** | **67** | **56.3** | **21.5** | **34.5** | **20.3** | **35.3** | **23.4** |
| Mean size of all fibers (kb) | 51.1 | 40.15 | 57.52 | 59.74 | 108.1 | 74.88 | 82.19 | 92.2 |
| Number of replication eyes | 31 | 52 | 143 | 256 | 182 | 311 | 286 | 409 |
| Mean Eye Length (EL) (kb) | 2.25 | 2.26 | 6.62 | 11.43 | 3.8 | 6.48 | 5.37 | 6.7 |
| Median Eye Length (kb) | 1.2 | 1.3 | 3.5 | 7.3 | 2.2 | 2.2 | 2.2 | 2.9 |
| Number of Eye to Eye Distances (ETED) | 7 | 24 | 71 | 147 | 93 | 170 | 157 | 286 |
| Mean ETED length (kb) | 13.23 | 13.41 | 14.61 | 17.87 | 30.3 | 20.30 | 23.55 | 15.8 |
| Median ETED length (kb) | 11.3 | 8.6 | 12.5 | 13.5 | 19.7 | 14.1 | 17.8 | 11 |
| Number of replication forks | 66 | 110 | 356 | 704 | 393 | 680 | 606 | 930 |
| **Fork Density (forks/100 kb)** | **1.42** | **2.91** | **3.14** | **5.51** | **2.51** | **4.61** | **3.34** | **5.25** |
| **∆Yap/Mock fork density** | **2.1** | | **1.8** | | **1.8** | | **1.6** | |
