# Supplementary material for: A non-transcriptional function of Yap regulates the DNA replication program in Xenopus laevis
Source: eLife. 2022 Jul 15;11:e75741. doi: 10.7554/eLife.75741 (PMC9328763; doi:10.7554/eLife.75741)
Supplement: Supplementary file 2. — Extended DNA combing data to Figure 4 of 2 independent experiments, Replicate 1 and Replicate 2. [file elife-75741-supp2.docx]

**Supplementary File 2**

**Depletion of Rif1 increases replication origin firing in *Xenopus* egg extracts**

Extended DNA combing data to Figure 4

2 independent experiments, Replicate 1 and Replicate 2

**Replicate 1**

| Time | 50 min | | 60 min | | 75 min | | 90 min | | 105 min | |
| --- | --- | --- | --- | --- | --- | --- | --- | --- | --- | --- |
| condition | ∆Mock | ∆Rif1 | ∆Mock | ∆Rif1 | ∆Mock | ∆Rif1 | ∆Mock | ∆Rif1 | ∆Mock | ∆Rif1 |
| Analysed DNA (kb) | 57317.4 | 33913.6 | 50565.5 | 63104.9 | 34450.7 | 55022.6 | 32863.5 | 22546.7 | 24290 | 34122.3 |
| Replicated DNA(kb) | 1376.3 | 6234 | 1778.9 | 14876.4 | 2705.7 | 18221.1 | 6299 | 9372.4 | 7151.3 | 17207.5 |
| **Replicated fraction** | **0.024** | **0.183** | **0.0351** | **0.235** | **0.0785** | **0.331** | **0.1916** | **0.415** | **0.294** | **0.504** |
| **∆Rif1/Mock Replication** | **7.65** | | **6.70** | | **4.21** | | **2.17** | | **1.713** | |
| Number of analysed fibers | 482 | 352 | 673 | 690 | 366 | 652 | 288 | 258 | 352 | 349 |
| Number of fully replicated fibers | 2 | 15 | 6 | 29 | 33 | 9 | 11 | 14 | 34 | 15 |
| Number of unreplicated fibers | 393 | 133 | 548 | 186 | 171 | 117 | 111 | 54 | 52 | 41 |
| **% of unreplicated fibers** | **81.5** | **37.8** | **81.4** | **27** | **46.7** | **17.9** | **38.5** | **20.9** | **14.8** | **11.7** |
| Mean size of all fibers (kb) | 118.9 | 96.3 | 75.1 | 91.4 | 94.1 | 84.4 | 114.1 | 87.4 | 69.0 | 97.8 |
| Number of replication forks | 353 | 1180 | 455 | 2600 | 593 | 3628 | 1089 | 1469 | 1357 | 2779 |
| **Fork Density (forks/100 kb)** | **0.615** | **3.479** | **0.899** | **4.120** | **1.721** | **6.593** | **3.313** | **6.515** | **5.586** | **8.144** |
| **∆Rif1/Mock fork density** | **5.65** | | **4.58** | | **3.83** | | **1.97** | | **1.46** | |

**Replicate 2**

| Time | 60 min | | 75 min | | 90 min | | 105 min | |
| --- | --- | --- | --- | --- | --- | --- | --- | --- |
| condition | ∆Mock | ∆Rif1 | ∆Mock | ∆Rif1 | ∆Mock | ∆Rif1 | ∆Mock | ∆Rif1 |
| Analysed DNA (kb) | 4727.4 | 32949.1 | 24942.5 | 38001.2 | 40710.0 | 51519.9 | 47109.9 | 50042.1 |
| Replicated DNA (kb) | 527.4 | 2552.9 | 964.2 | 3853.5 | 4824.0 | 17415.5 | 12500.3 | 26768.7 |
| **Replicated fraction** | **0.011** | **0.077** | **0.039** | **0.101** | **0.118** | **0.338** | **0.265** | **0.5349** |
| **∆Rif1/Mock Replication** | **6.94** | | **2.62** | | **2.85** | | **2.02** | |
| Number of analysed fibers | 371 | 402 | 352 | 525 | 675 | 917 | 780 | 1320 |
| Number of fully replicated fibers | 1 | 23 | 4 | 5 | 56 | 78 | 152 | 367 |
| Number of unreplicated Fibers | 307 | 218 | 264 | 337 | 220 | 143 | 147 | 116 |
| **% of unreplicated Fibers** | **82.7** | **54.2** | **75** | **64.2** | **32.6** | **15.6** | **18.8** | **8.7** |
| Average size of all fibers (kb) | 127.4 | 81.9 | 70.9 | 72.4 | 60.3 | 56.2 | 60.4 | 37.9 |
| Number of replication fork | 241 | 692 | 297 | 922 | 1215 | 3268 | 2627 | 4067 |
| **Fork Density (forks/100 kb)** | **0.51** | **2.1** | **1.19** | **2.42** | **2.98** | **6.34** | **5.58** | **8.13** |
| **∆Rif1/Mock fork density** | **4.12** | | **2.034** | | **2.13** | | **1.46** | |
